# Supplementary material for: Nanopore-Based Profiling of PEGylation in Nucleic Acid Therapeutics
Source: J Am Chem Soc. 2026 Jul 27;148(31):33980–9. doi: 10.1021/jacs.6c12398 (PMC13474927; doi:10.1021/jacs.6c12398)
Supplement: Supplementary file 1 [file ja6c12398_si_001.pdf]

Supplementary Material for

Nanopore-based profiling of PEGylation in nucleic acid therapeutics

AUTHOR NAMES

Gerardo Patiño Guillén[1,‡], Thieme T. Schmidt[1,‡], Jeremy J. Baumberg[1], Jack W. Szostak[2], Ulrich F. Keyser[1,*], Filip Bošković[2,*]

AUTHOR ADDRESS

[1]Cavendish Laboratory, University of Cambridge, Cambridge, CB3 0HE, UK

[2]Howard Hughes Medical Institute, Department of Chemistry, The University of Chicago, Chicago, Illinois, 60637, USA

‡These authors contributed equally.

*Corresponding authors: filipboskovic@uchicago.edu; ufk20@cam.ac.uk

This PDF file includes:

- → Figures S1 to S16
- → Tables S1 to S9

Figure S1.

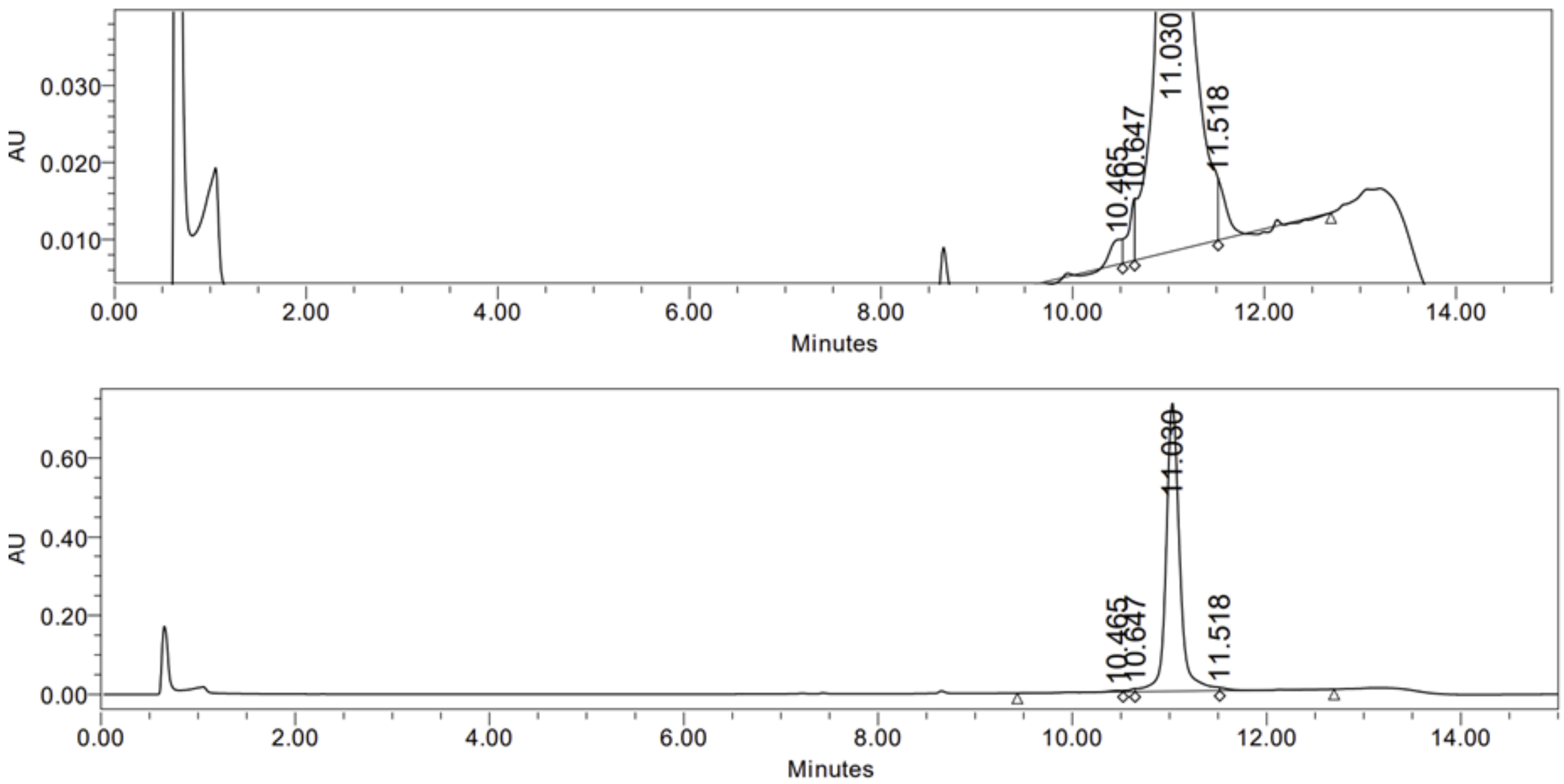

Figure S1. HPLC of PEGylated RNA - pegaptanib. Peak characterization indicates 98.01% of the conjugated pegaptanib, as it resolves the PEGylated RNA population from the non-PEGylated distribution.

Figure S2.

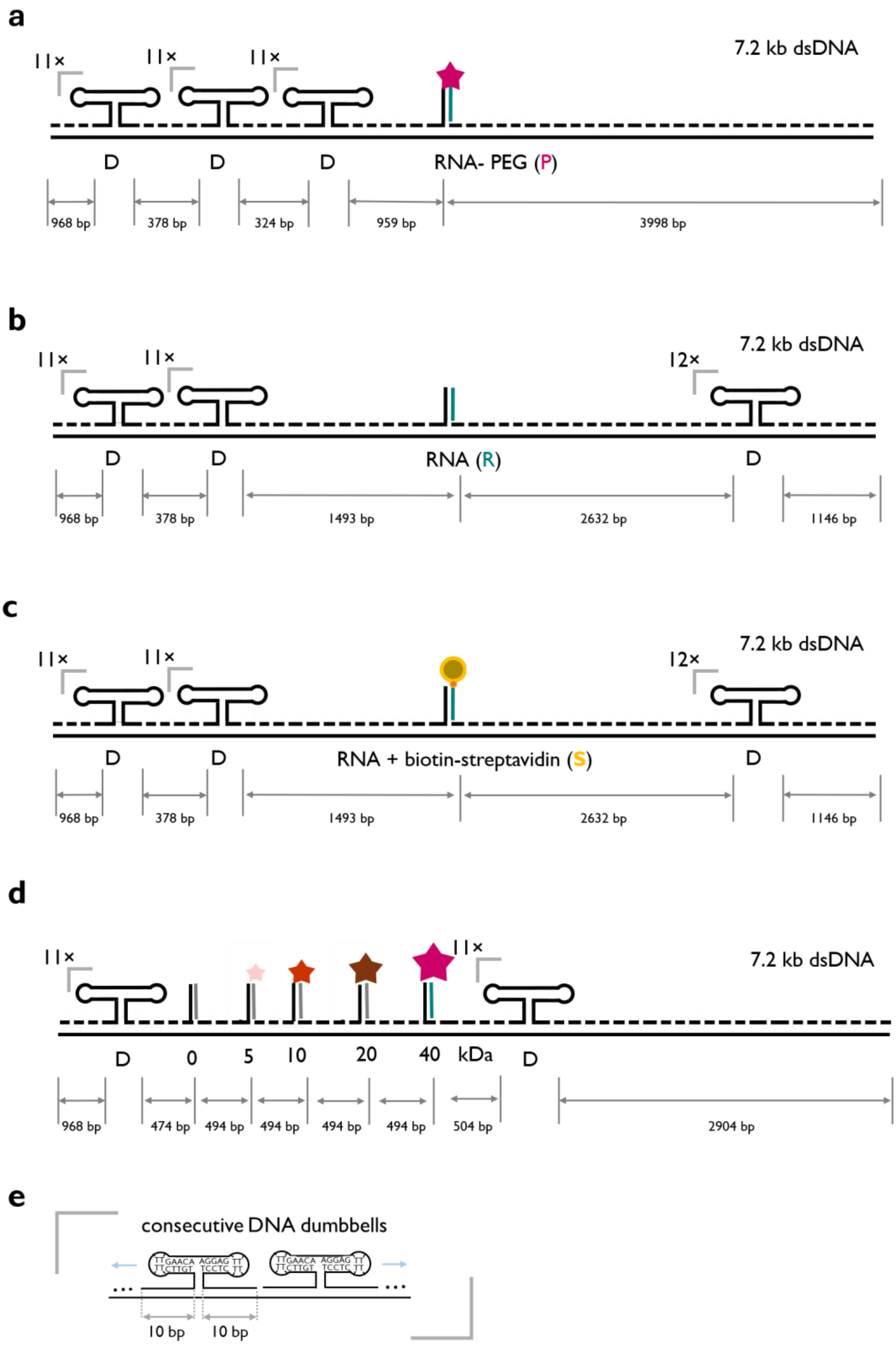

Figure S2. Detailed designs of DNA nanostructure holding **a** PEGylated pegaptanib ‘P’, **b** non-PEGylated pegaptanib ‘R’ and **c** biotinylated RNA ‘S’, **d** 0, 5, 10, 20 and 40 kDa PEG-oligonucleotide conjugates. **e** Detailed design of the DNA dumbbells featured in all nanostructures.

Figure S3.

a

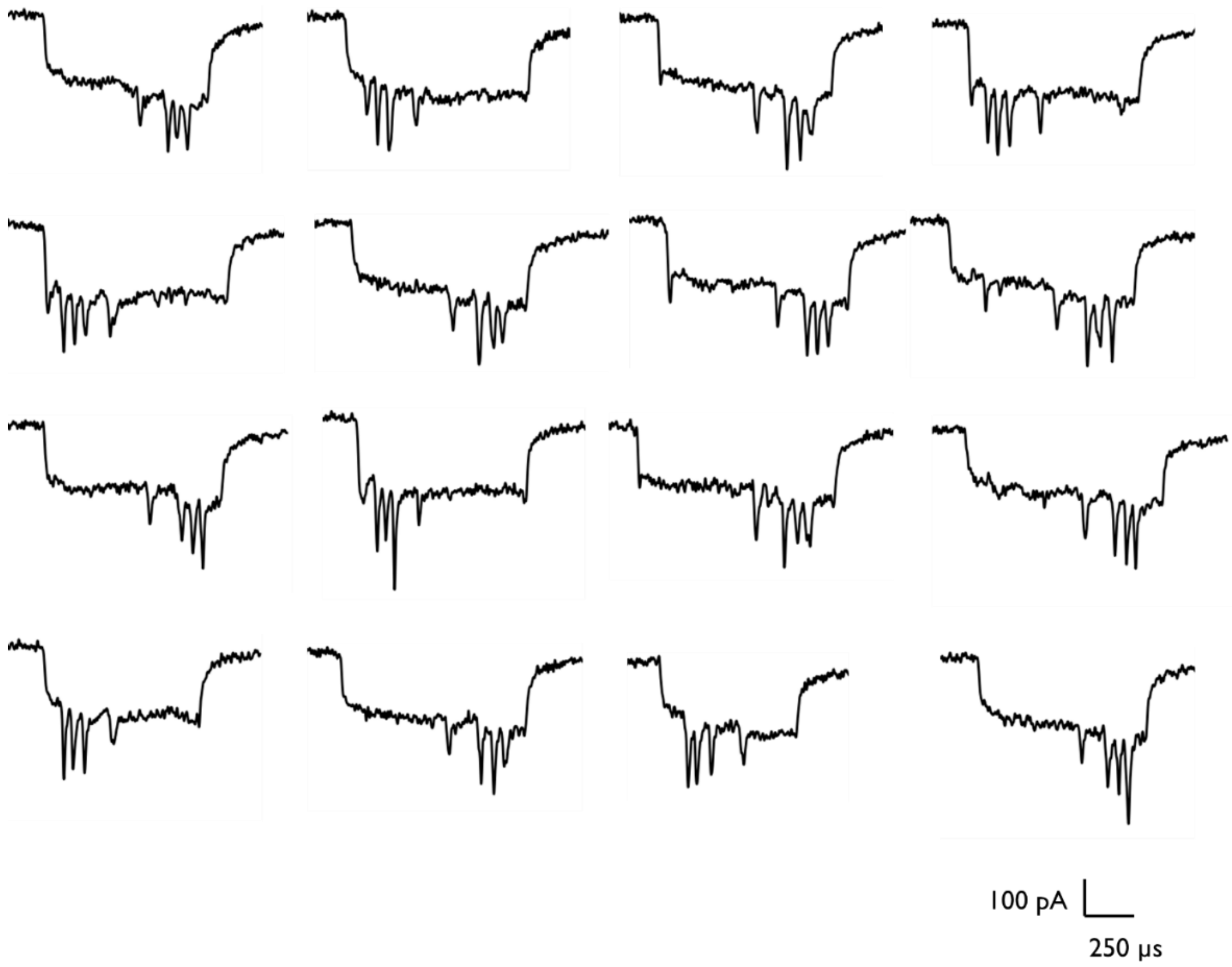

b

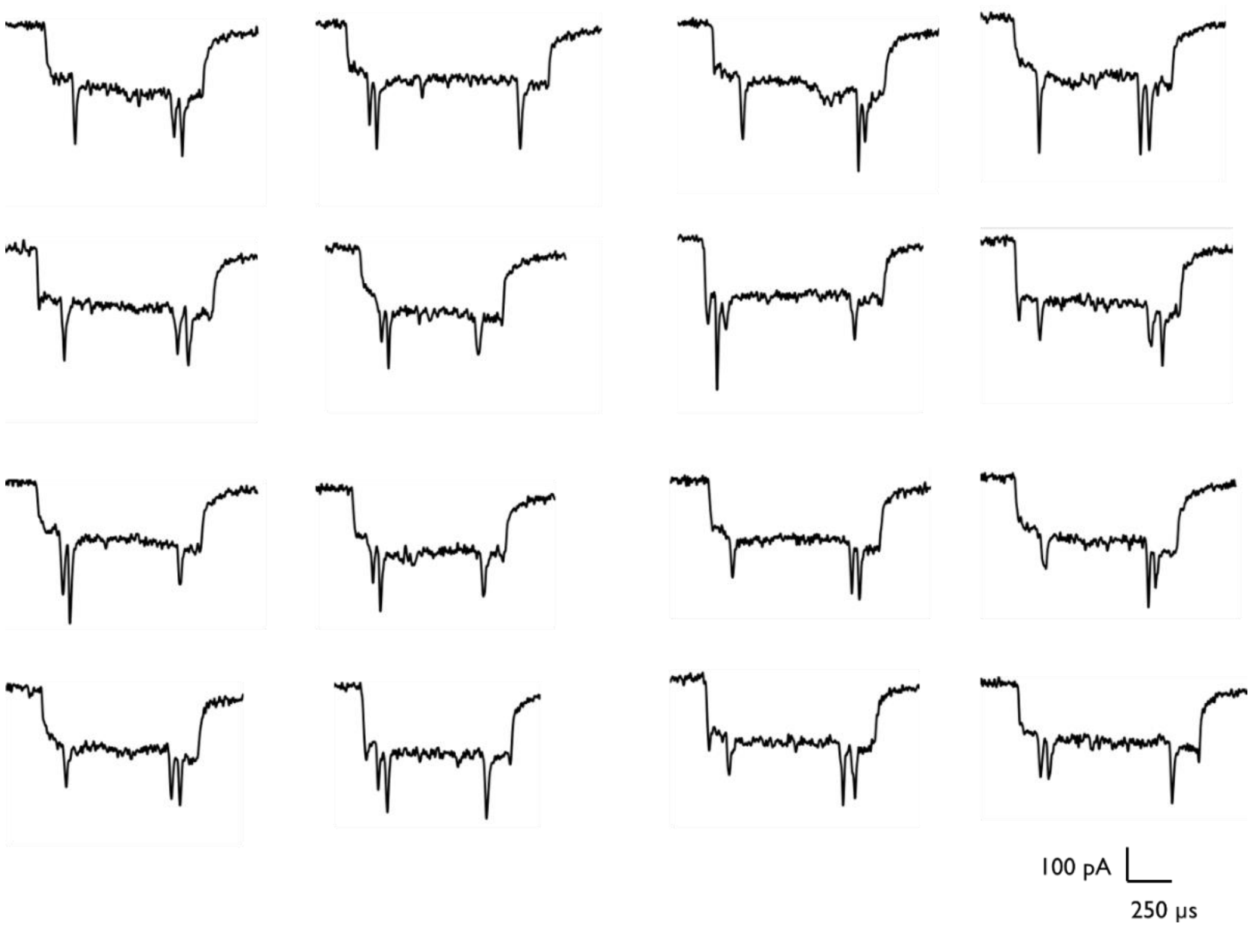

Figure S3. a Raw ionic current traces of unfolded DNA nanostructure holding PEGylated pegaptanib translocating through nanopore and **b** raw ionic current traces of unfolded DNA nanostructure holding RNA aptamer translocating through nanopore.

Figure S4.

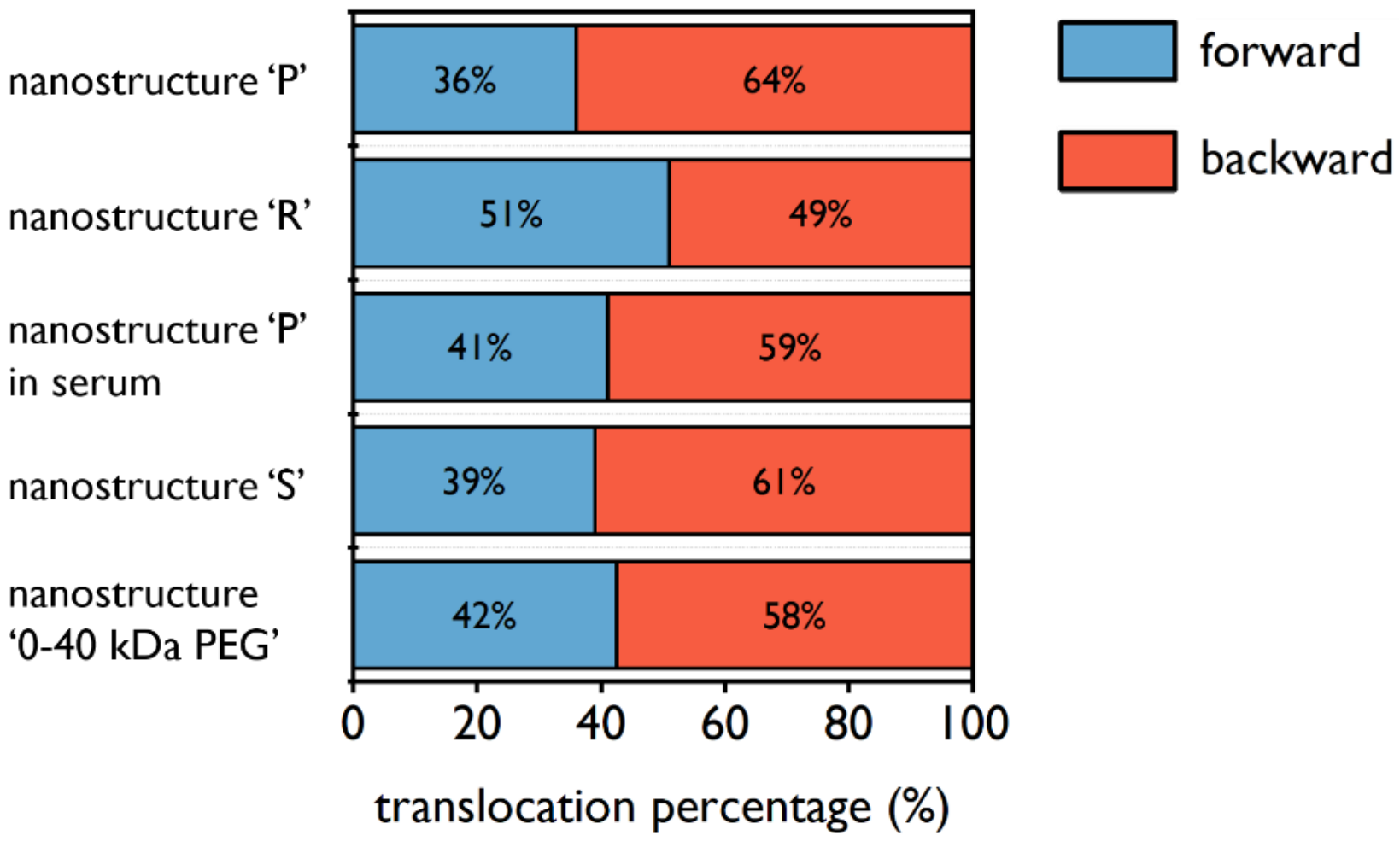

Figure S4. Directionality of nanostructure translocation. We show the percentage of unfolded translocation events that occur in each direction (forward 0→7.2 kbp and backward 7.2→0 kbp) for the different nanostructures sensed.

Figure S5.

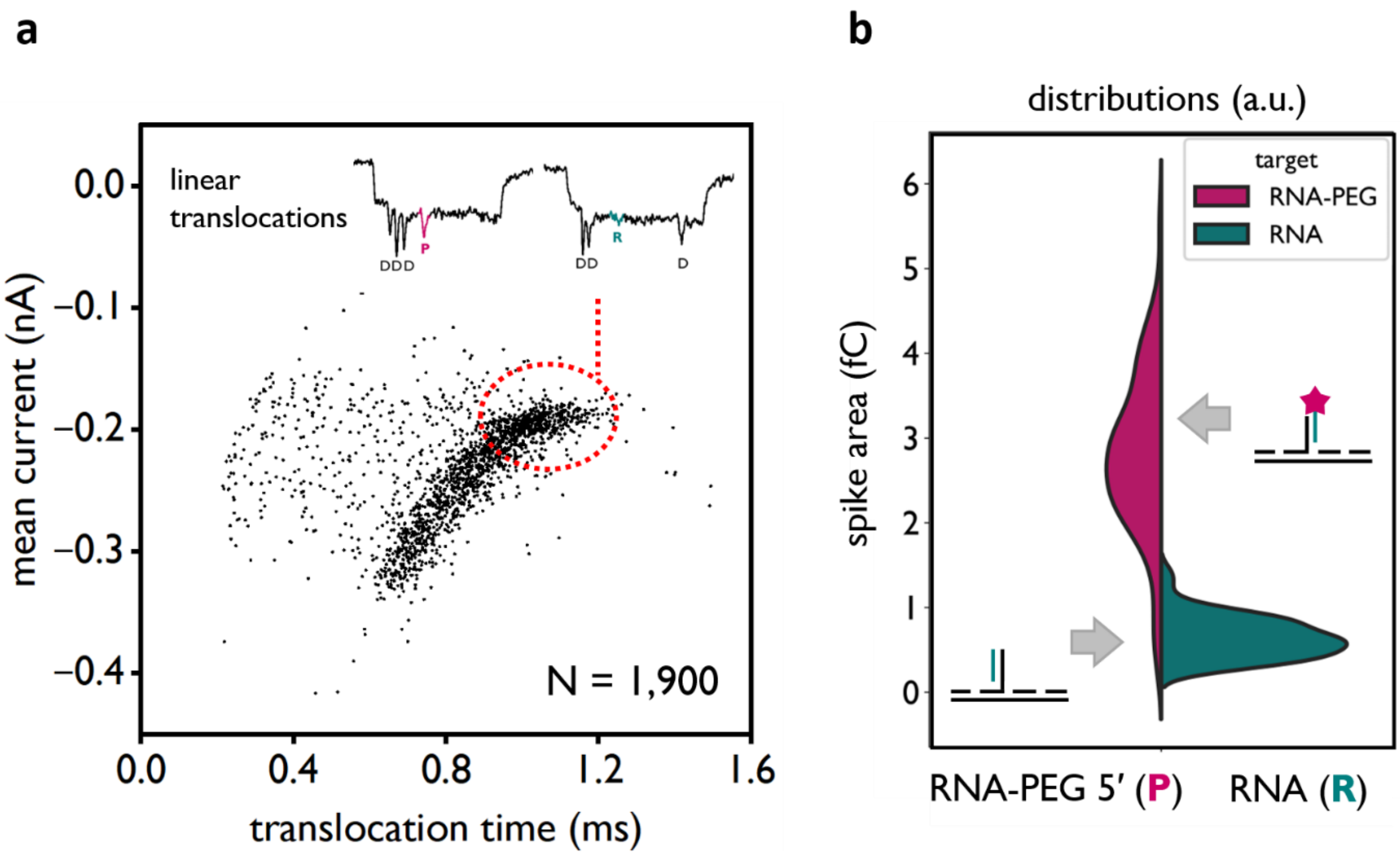

Figure S5. a Nanopore translocation observables enable the identification of linear translocation events. Unfolded nanostructures passing through the nanopore exhibit longer translocation times and smaller mean current drops. These features allow discrimination from folded translocation events, in which the nanostructure adopts a compact conformation during translocation, compromising the recognition of the nanostructure's features. **b** In linear translocation events, the current spike area is used to quantify PEGylation. We compared the current spike area of 'P' and 'R', which correspond to the spike of PEGylated and non-PEGylated pegaptanib, respectively. This enabled quantitative distinction between both aptamer species.

Figure S6.

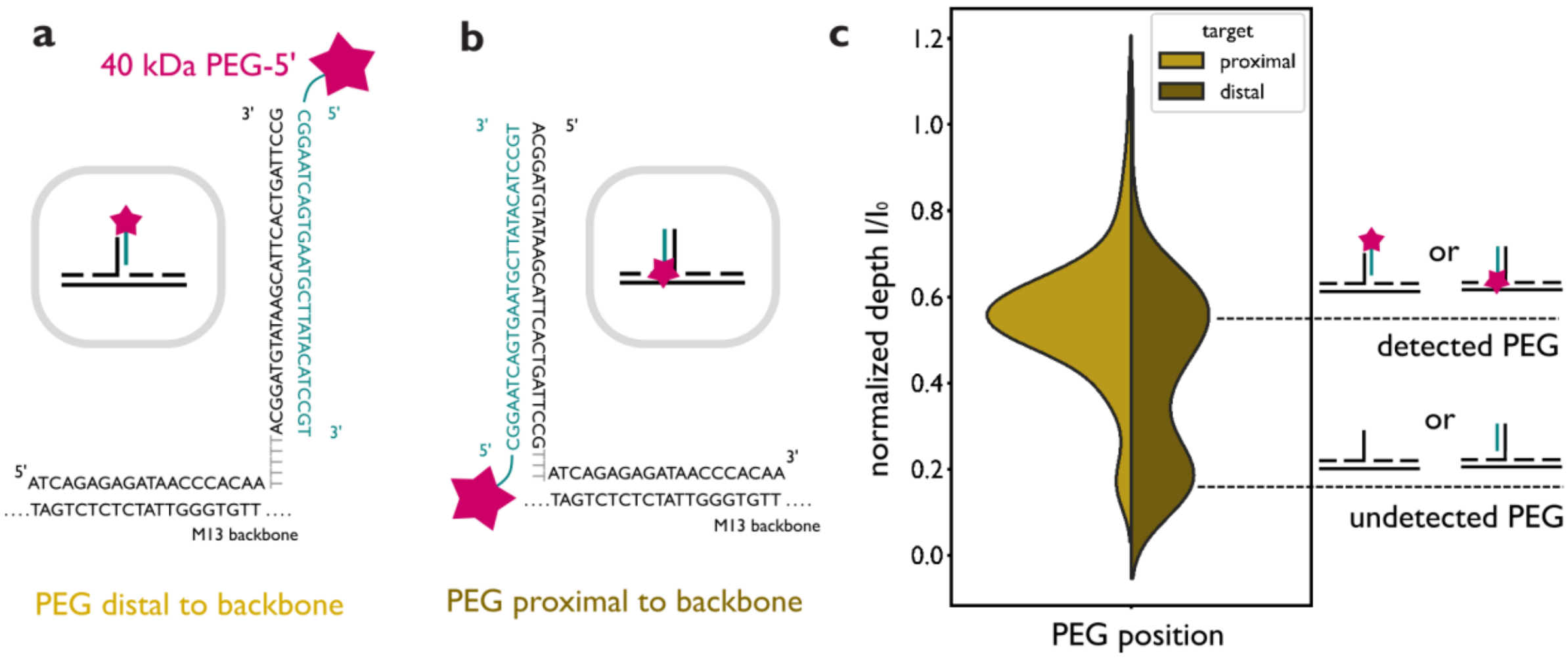

Figure S6. Optimization of pegaptanib capture in the DNA nanostructure. We observed that the proximity of 40 kDa PEG to the DNA nanostructure's backbone played a key role in capture efficiency. **a** Shows PEG distal to the backbone, where the PEGylated 5′ end of pegaptanib is pointing to the outside of the nanostructure and the DNA overhang has 5 'dT' as spacer. **b** Shows PEG proximal to the backbone, where the PEGylated 5′ end of pegaptanib is pointing towards the backbone and only 3 'dT' separate the overhang. **c** With the distal conformation, ≈94% of nanopore translocations showed PEGylated pegaptanib, which agrees with HPLC data. In contrast, we detected only ≈61% PEGylated pegaptanib with the proximal conformation. We attribute this difference to the steric hindrance of 40 kDa PEG to the DNA nanostructure backbone in the proximal conformation. Despite the different conformations influence capture efficiency, the normalized spike depth is not significantly affected. We utilized the distal conformation for PEGylation characterization.

Figure S7

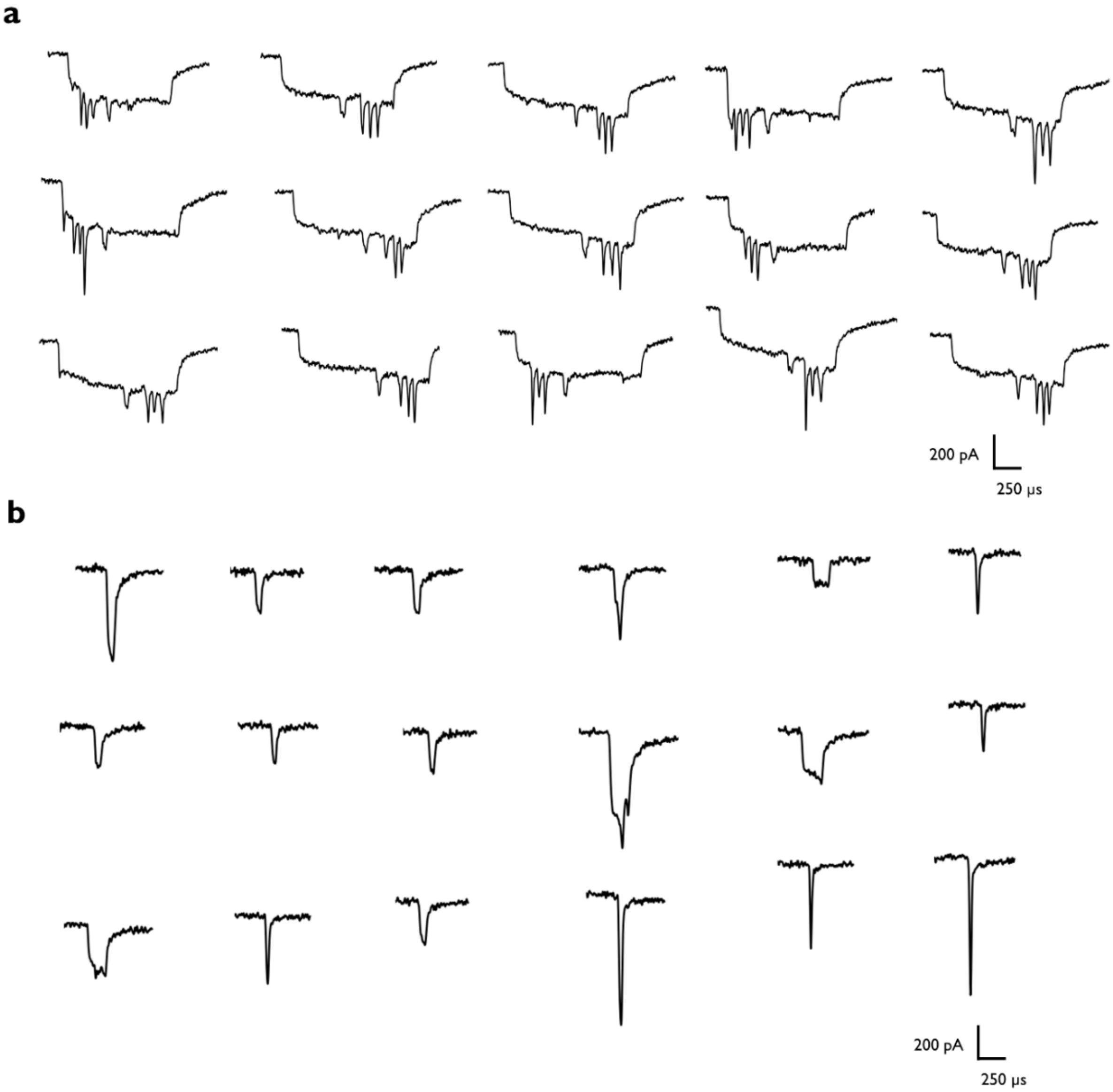

Figure S7. Raw nanopore ionic current traces of **a** unfolded DNA nanostructure assembled in the presence of horse serum holding PEGylated pegaptanib, and **b** non-specific translocations attributed to complex background.

Figure S8.

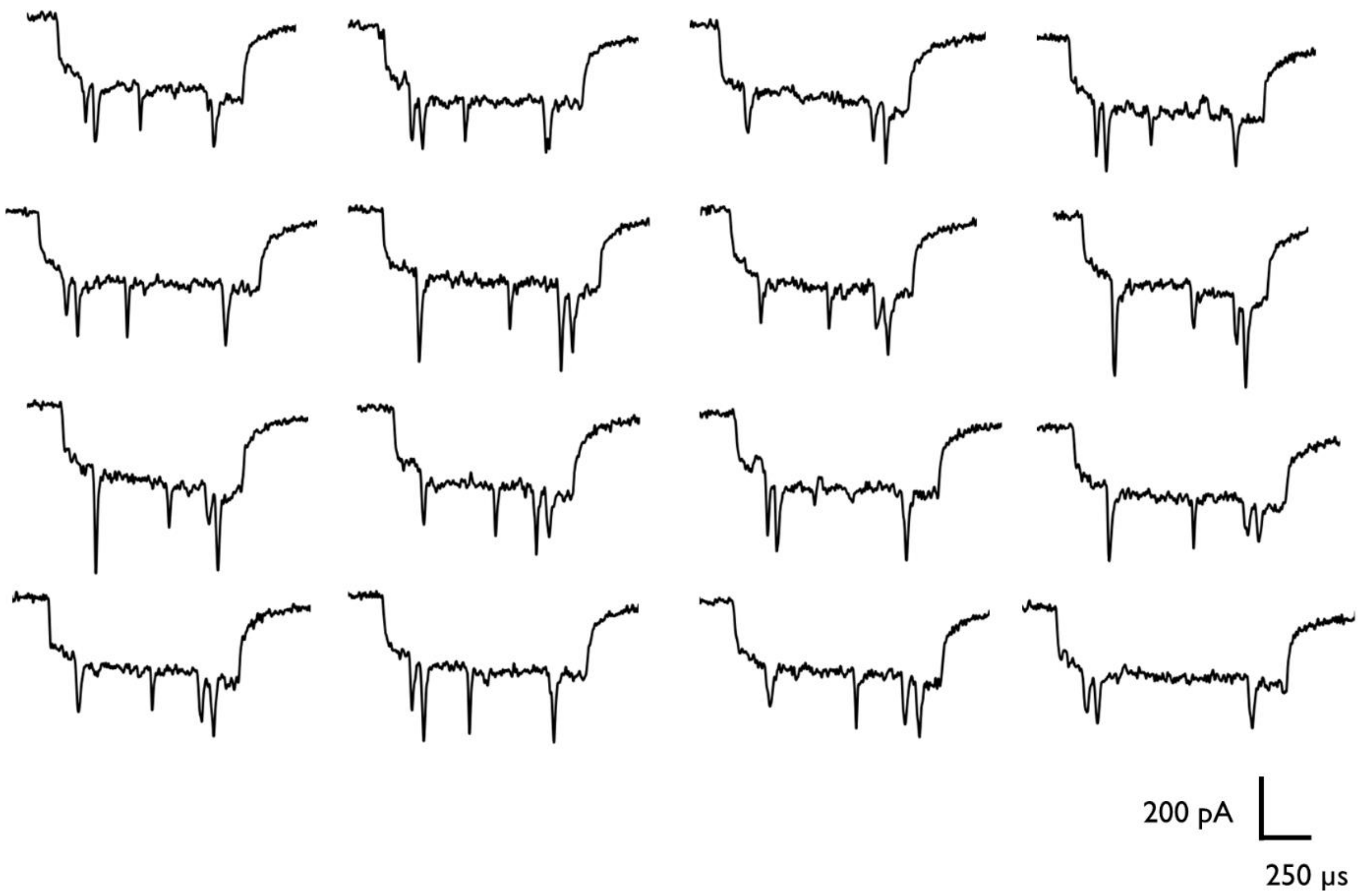

Figure S8. Raw nanopore ionic current traces of linear, unfolded DNA nanostructures holding RNA aptamer with a bound streptavidin-biotin complex.

Figure S9.

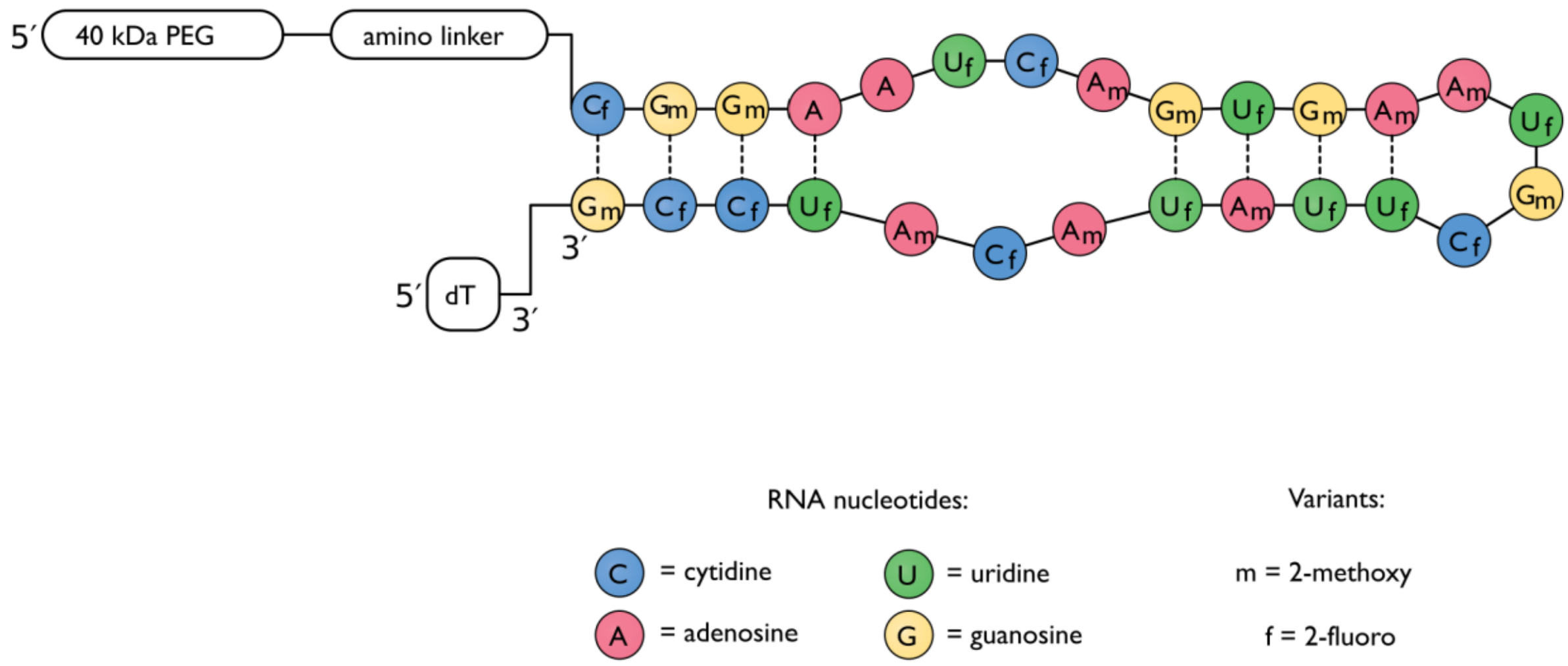

Figure S9. Pegaptanib sequence, indicated backbone and terminal modifications.

Figure S10

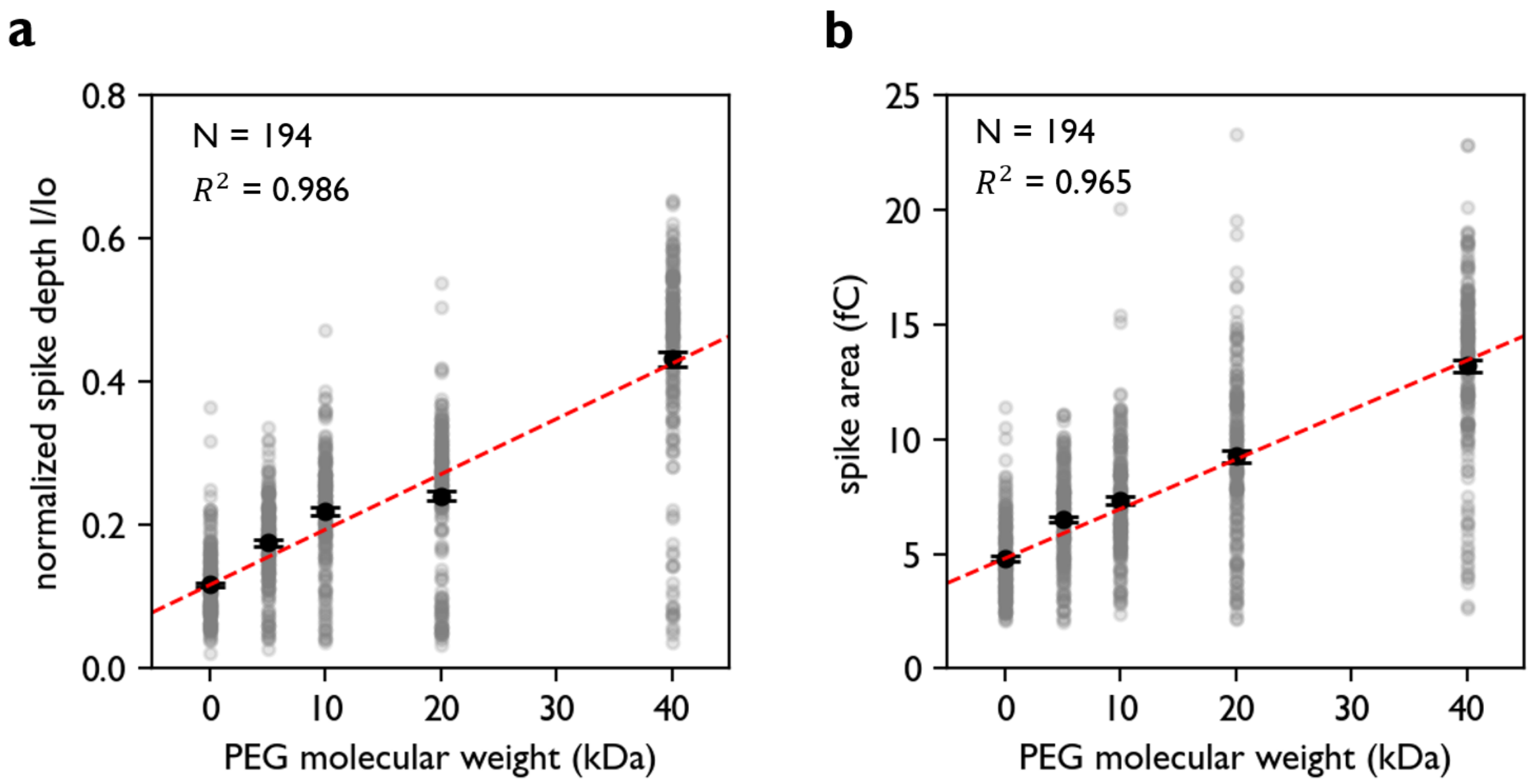

Figure S10. Correlation of PEG molecular weight (kDa) with **a** the normalized spike depth *I/Io* and **b** the spike area. The measurements correspond to a single nanostructure holding PEG with molecular weights of 0, 5, 10, 20 and 40 kDa.

Figure S11.

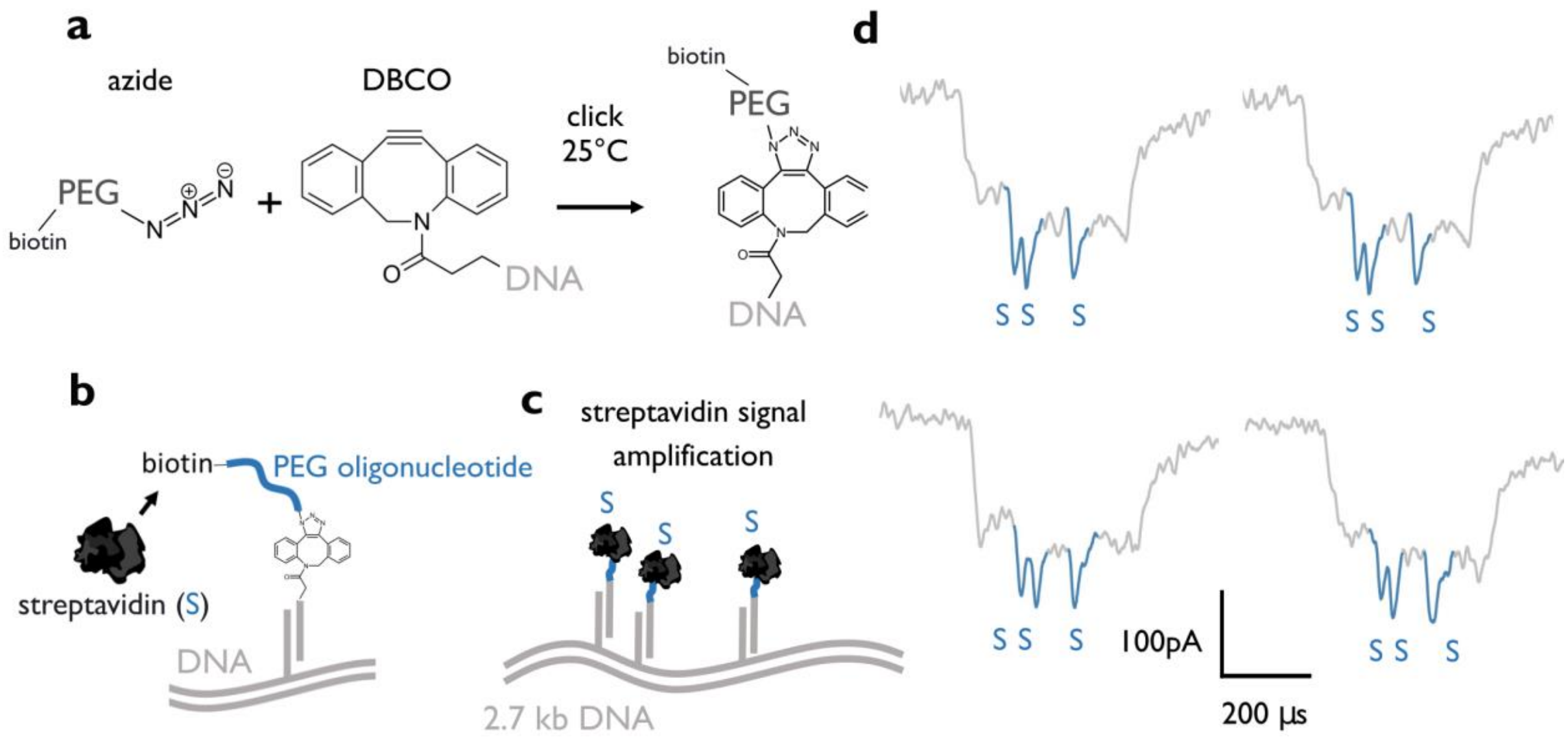

Figure S11. Streptavidin detection *via* biotin-PEG binding in nanopores. **a** azide-activated PEG3 oligonucleotide (Merk, 762024) and 3′-DBCO activated DNA oligonucleotide are clicked. PEG holds a biotin group in one end. **b** We capture PEG-DNA in a 2.7 kb DNA nanostructure and **c** streptavidin binds to the biotin group of PEG. **d** Streptavidin attachment produces a prominent downward current spike in nanopore sensing further validating PEG incorporation to the nanostructure.

Figure S12.

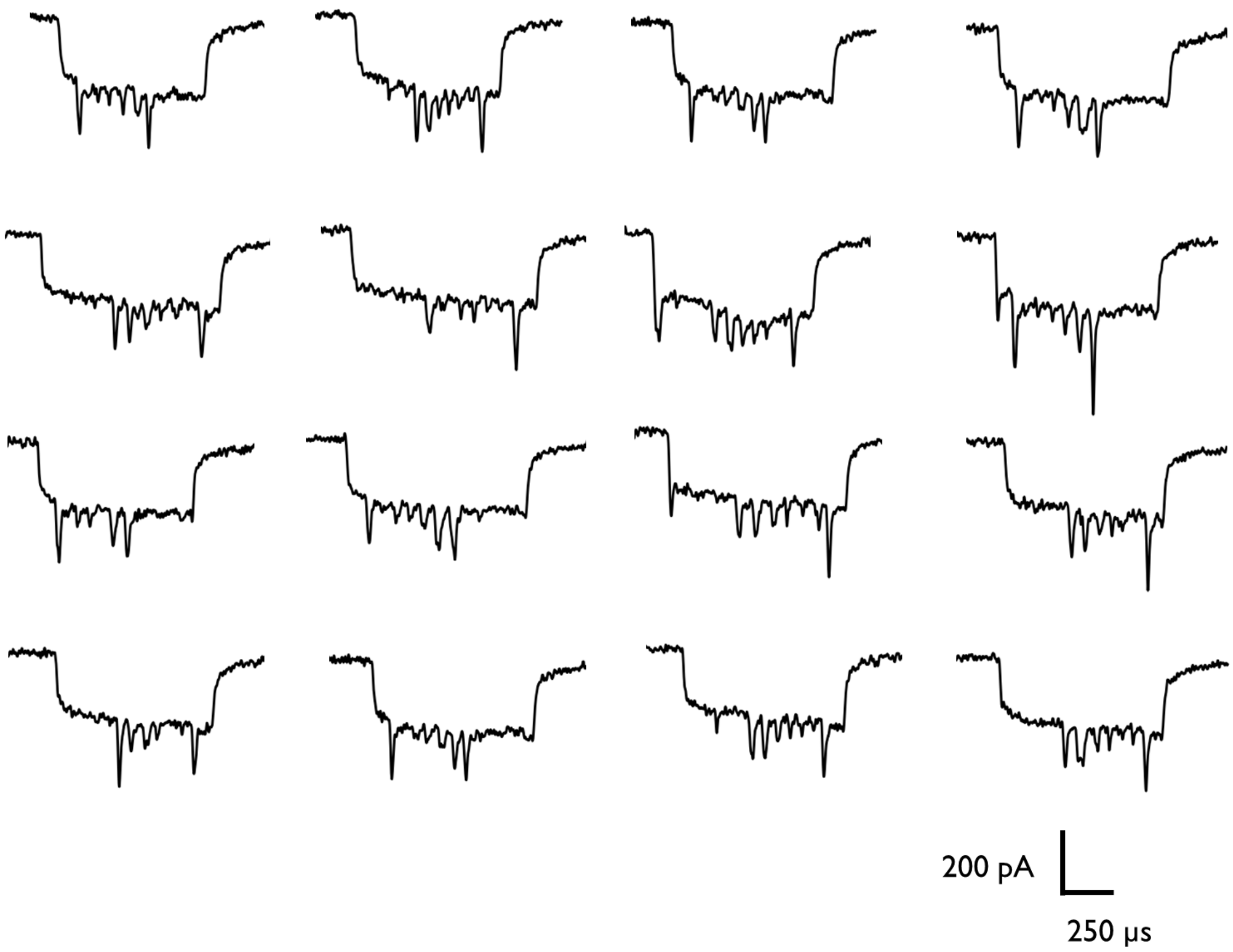

Figure S12. Raw ionic current traces of unfolded DNA nanostructure nanopore events holding PEGylated oligonucleotide conjugates (0,5, 10, 20 kDa) and pegaptanib (40 kDa PEG-RNA).

Figure S13.

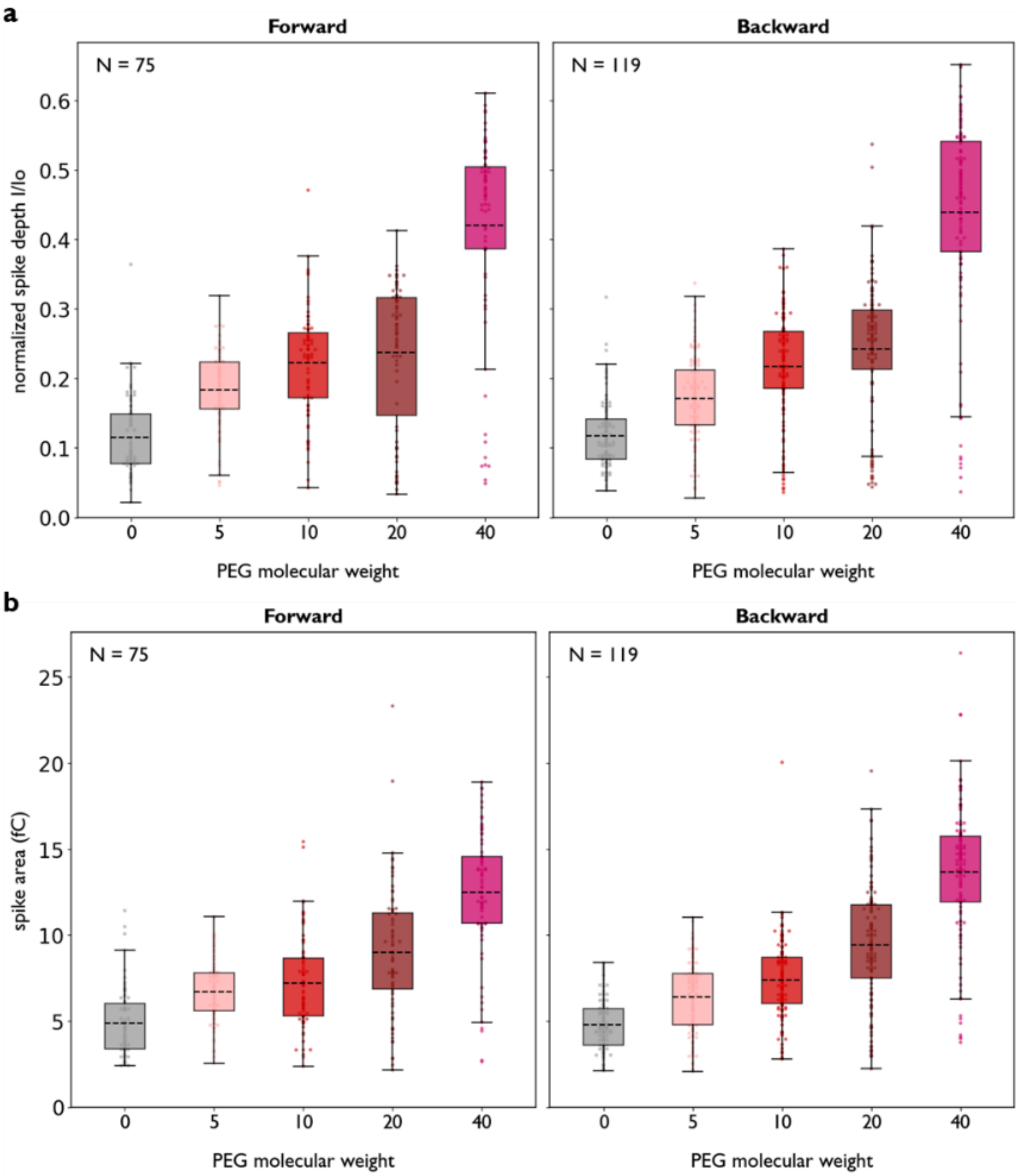

Figure S13. Translocation direction dependence of current spike observables. **a** Normalized spike depth for events separated by translocation direction. **b** Spike area for events separated by translocation direction. A total of 75 forward and 119 backward unfolded translocations were detected.

Figure S14.

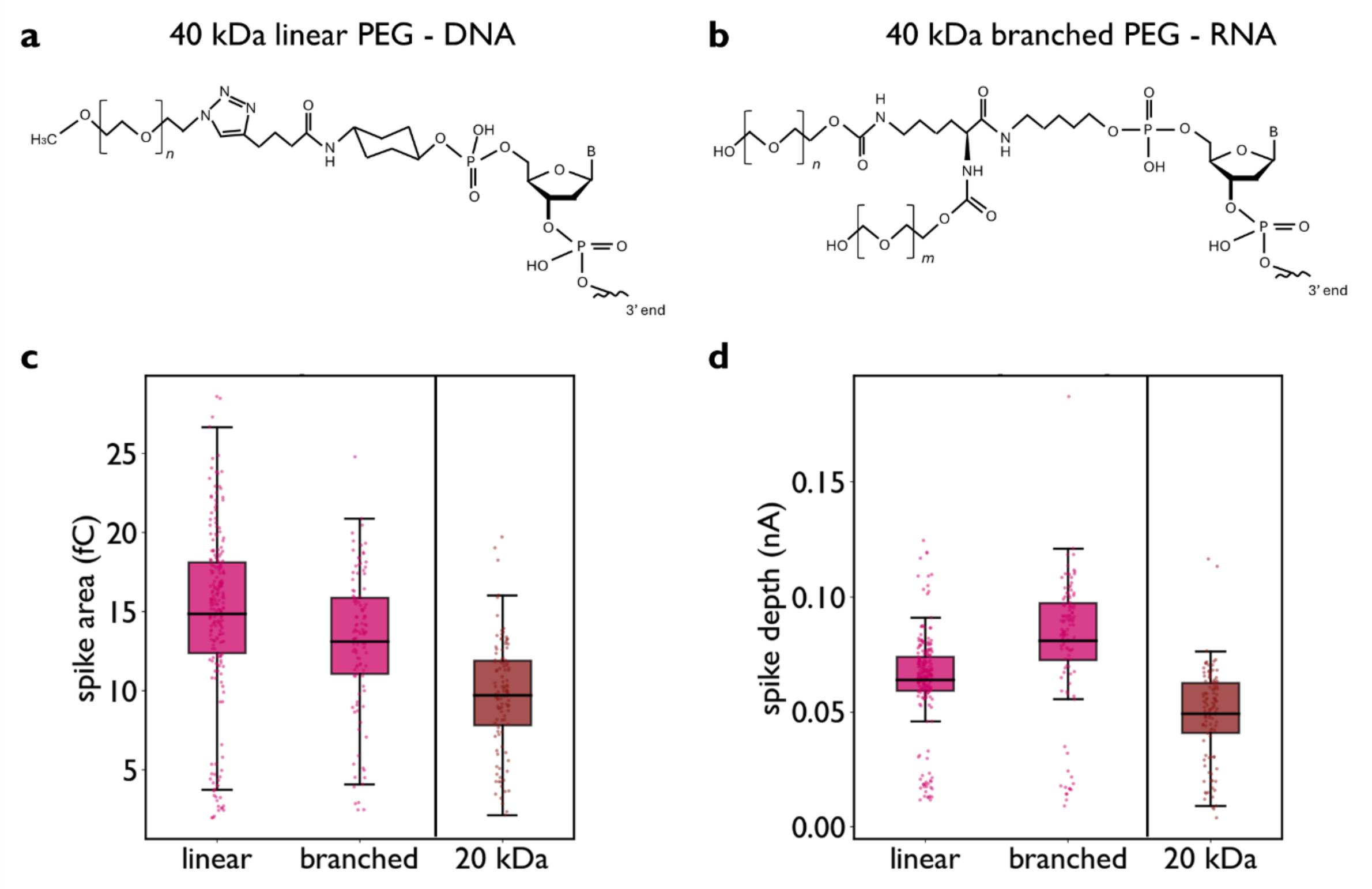

Figure S14. For the assessment of PEG molecular weight of in 20 and 40 kDa PEG mixtures, we use **a** 40 kDa linear PEG – DNA conjugate purchased from biomers (*mPEG 40kDa, mPEG 40000*), instead of the **b** 40 kDa branched PEG bound to the RNA aptamer (pegaptanib), to prevent hybridization biases. **c** We showed that the spike area provides better discrimination between the nanostructure holding 40 kDa linear PEG and the one with 20 kDa PEG, due to reduced overlap in the distributions.

Figure S15.

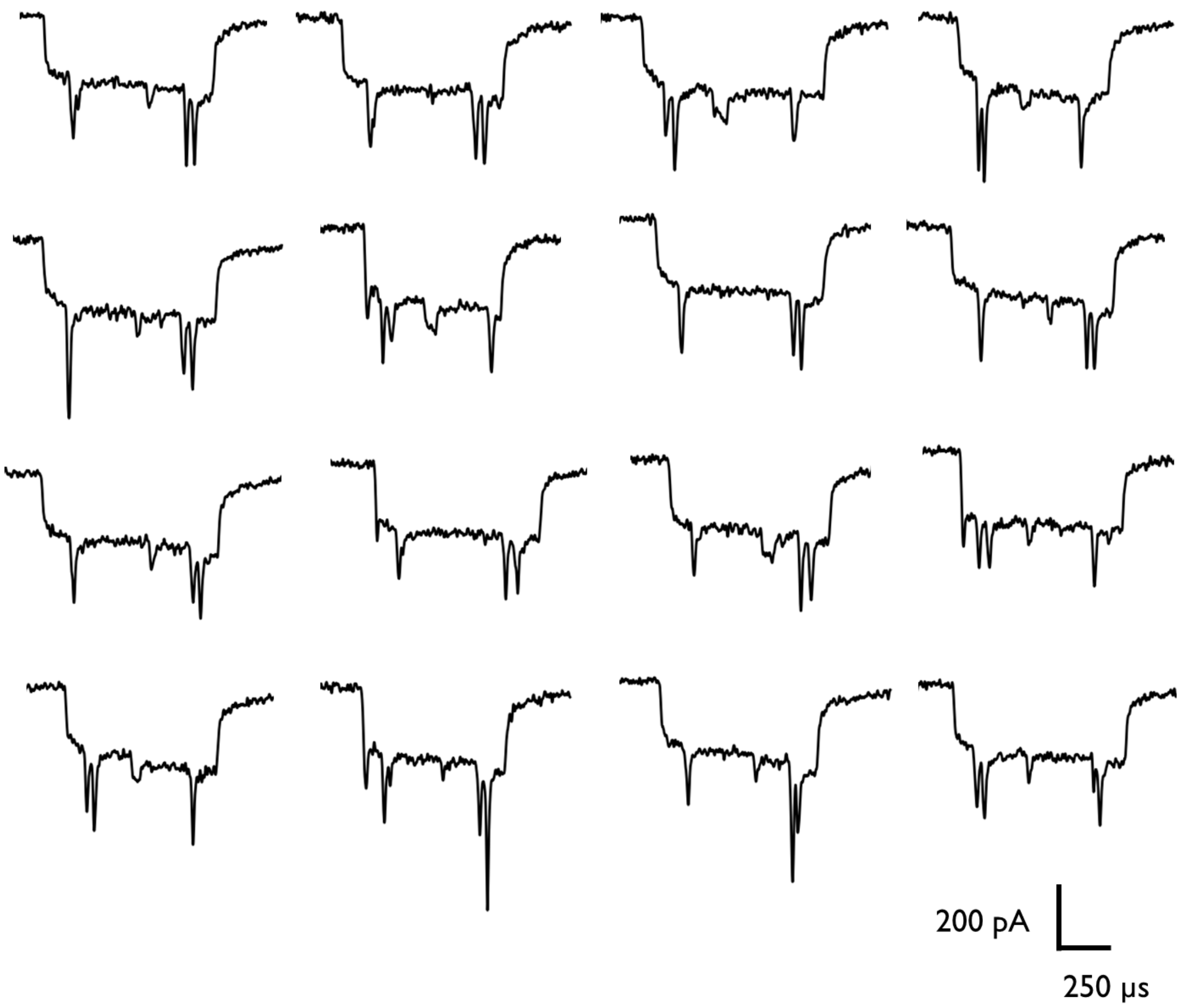

Figure S15. Raw nanopore ionic current traces of unfolded DNA nanostructure holding a mix of 20 and 40 kDA PEG-DNA conjugates.

Figure S16.

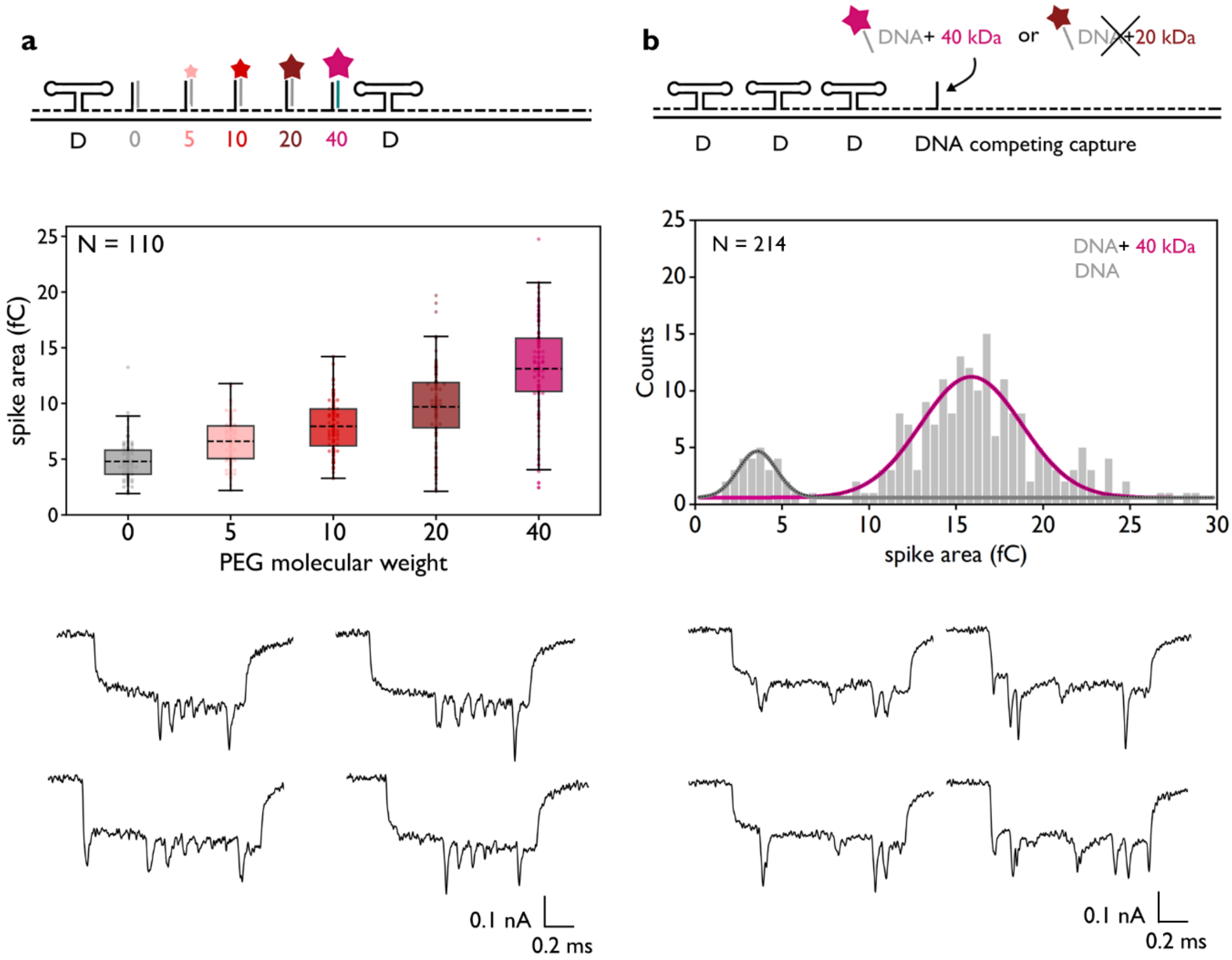

Figure S16. DNA nanostructure carrying 0, 5, 10, 20, and 40 kDa PEG conjugates as spike-area references for PEG molecular-weight assignment (**a**). In the same nanopore measurement, **b** we included a nanostructure which captured only the 40 kDa PEG conjugate, after removing the 20 kDa PEG conjugate from the mixture. In the spike area distribution, the population ascribed to 20 kDa PEG in Figure 4f, is no longer depicted.

Table S1.

Sequence of PEGylated RNA aptamer, pegaptanib.

Oligo number	Sequence (5′→ 3′)
1	5′-modification (40kDa)-CGGAAUCAGUGAAUGCUUAUACAUCCGU

Table S2.

Sequence of 1) PEGylated RNA aptamer, 2) non-PEGylated RNA aptamer, 3) capture DNA oligonucleotide.

Oligo number	Sequence (5′→ 3′)
1	5′-modification (40kDa)-CGGAAUCAGUGAAUGCUUAUACAUCCGU
2	CGGAAUCAGUGAAUGCUUAUACAUCCGU
3	ATCAGAGAGATAACCCACAAttttttACGGATGTATAAGCATTCACTGATTCCG

Table S3.

Sequence of DNA oligonucleotides used for assembly of dsDNA nanostructure used for capture of PEGylated pegaptanib 'P'.

Oligonucleotide / **Dumbbell** / **Overhang**

Oligo number	Sequence (5′→ 3′)
1	TTTTCGTAATCATGGTCATAGCTGTTTCCTGTGTGAAATTGTTATC
2	CGCTCACAATTCCACACAACATACGAGCCGGAAGCATA
3	AAGTGTAAAGCCTGGGGTGCCTAATGAGTGAGCTAACT
4	CACATTAATTGCGTTGCGCTCACTGCCCGCTTTCCAGT
5	CGGGAAACCTGTCGTGCCAGCTGCATTAATGAATCGGC
6	CAACGCGCGGGGAGAGGCGGTTTGCGTATTGGGCGCCA
7	GGGTGGTTTTTCTTTTCACCAGTGAGACGGGCAACAGC
8	TGATTGCCCTTCACCGCCTGGCCCTGAGAGAGTTGCAG
9	CAAGCGGTCCACGCTGGTTTGCCCCAGCAGGCGAAAAT
10	CCTGTTTGATGGTGGTTCCGAAATCGGCAAAATCCCTT
11	ATAAATCAAAAGAATAGCCCGAGATAGGGTTGAGTGTT
12	GTTCCAGTTTGGAACAAGAGTCCACTATTAAAGAACGT
13	GGACTCCAACGTCAAAGGGCGAAAAACCGTCTATCAGG
14	GCGATGGCCCACTACGTGAACCATCACCCAAATCAAGT
15	TTTTTGGGGTCGAGGTGCCGTAAAGCACTAAATCGGAA
16	CCCTAAAGGGAGCCCCCGATTTAGAGCTTGACGGGGAA
17	AGCCGGCGAACGTGGCGAGAAAGGAAGGGAAGAAAGCG
18	AAAGGAGCGGGCGCTAGGGCGCTGGCAAGTGTAGCGGT
19	CACGCTGCGCGTAACCACCACACCCGCCGCGCTTAATG
20	CGCCGCTACAGGGCGCGTACTATGGTTGCTTTGACGAG
21	CACGTATAACGTGCTTTCCTCGTTAGAATCAGAGCGGG
22	AGCTAAACAGGAGGCCGATTAAAGGGATTTTAGACAGG
23	AACGGTACGCCAGAATCCTGAGAAGTGTTTTTATAATC
24	AGTGAGGCCACCGAGTAAAAGAGTCTGTCCATCACGCA
25	AATTAACCGTTGTAGCAATACTTCTTTGATTAGTAATA
26	**ACATCACTTGTCCTCTTTTGAGGAACAAGTTTTCTTGTCCTGAGTAGA**
27	**AGAACTCAAATCCTCTTTTGAGGAACAAGTTTTCTTGTCTATCGGCCT**
28	**TGCTGGTAATTCCTCTTTTGAGGAACAAGTTTTCTTGTATCCAGAACA**
29	**ATATTACCGCTCCTCTTTTGAGGAACAAGTTTTCTTGTCAGCCATTGC**
30	**AACAGGAAAATCCTCTTTTGAGGAACAAGTTTTCTTGTACGCTCATGG**
31	**AAATACCTACTCCTCTTTTGAGGAACAAGTTTTCTTGTATTTTGACGC**
32	**TCAATCGTCTTCCTCTTTTGAGGAACAAGTTTTCTTGTGAAATGGATT**
33	**ATTTACATTGTCCTCTTTTGAGGAACAAGTTTTCTTGTGCAGATTCAC**
34	**CAGTCACACGTCCTCTTTTGAGGAACAAGTTTTCTTGTACCAGTAATA**
35	**AAAGGGACATTCCTCTTTTGAGGAACAAGTTTTCTTGTTCTGGCCAAC**

36	**AGAGATAGAATCCTCTTTTGAGGAACAAGTTTTCTTGTCCCTTCTGAC**
37	CTGAAAGCGTAAGAATACGTGGCACAGACAATATTTTTGAATGGCT
38	ATTAGTCTTTAATGCGCGAACTGATAGCCCTAAAACAT
39	CGCCATTAAAAATACCGAACGAACCACCAGCAGAAGAT
40	AAAACAGAGGTGAGGCGGTCAGTATTAACACCGCCTGC
41	AACAGTGCCACGCTGAGAGCCAGCAGCAAATGAAAAAT
42	CTAAAGCATCACCTTGCTGAACCTCAAATATCAAACCC
43	TCAATCAATATCTGGTCAGTTGGCAAATCAACAGTTGA
44	AAGGAATTGAGGAAGGTTATCTAAAATATCTTTAGGAG
45	AATATAATCCTGATTGTTTGGATTATACTTCTGAATAATGGAAGGG
46	**CACTAACAACTCCTCTTTTGAGGAACAAGTTTTCTTGTTAATAGATTA**
47	**GAGCCGTCAATCCTCTTTTGAGGAACAAGTTTTCTTGTTAGATAATAC**
48	**ATTTGAGGATTCCTCTTTTGAGGAACAAGTTTTCTTGTTTAGAAGTAT**
49	**TAGACTTTACTCCTCTTTTGAGGAACAAGTTTTCTTGTAAACAATTCG**
50	**ACAACTCGTATCCTCTTTTGAGGAACAAGTTTTCTTGTTTAAATCCTT**
51	**TGCCCGAACGTCCTCTTTTGAGGAACAAGTTTTCTTGTTTATTAATTT**
52	**TAAAAGTTTGTCCTCTTTTGAGGAACAAGTTTTCTTGTAGTAACATTA**
53	**TCATTTTGCGTCCTCTTTTGAGGAACAAGTTTTCTTGTGAACAAAGAA**
54	**ACCACCAGAATCCTCTTTTGAGGAACAAGTTTTCTTGTGGAGCGGAAT**
55	**TATCATCATATCCTCTTTTGAGGAACAAGTTTTCTTGTTTCCTGATTA**
56	**TCAGATGATGTCCTCTTTTGAGGAACAAGTTTTCTTGTGCAATTCATC**
57	TTAGAACCTACCATATCAAAATTATTTGCACGTAAAAC
58	AGAAATAAAGAAATTGCGTAGATTTTCAGGTTTAACGT
59	CAGATGAATATACAGTAACAGTACCTTTTACATCGGGA
60	GAAACAATAACGGATTCGCCTGATTGCTTTGAATACCA
61	AGTTACAAAATCGCGCAGAGGCGAATTATTCATTTCAA
62	TTACCTGAGCAAAAGAAGATGATGAAACAAACATCAAG
63	AAAACAAAATTAATTACATTTAACAATTTCATTTGAAT
64	TACCTTTTTTAATGGAAACAGTACATAAATCAATATAT
65	**GTGAGTGAATTCCTCTTTTGAGGAACAAGTTTTCTTGTAACCTTGCTT**
66	**CTGTAAATCGTCCTCTTTTGAGGAACAAGTTTTCTTGTTCGCTATTAA**
67	**TTAATTTTCCTCCTCTTTTGAGGAACAAGTTTTCTTGTCTTAGAATCC**
68	**TTGAAAACATTCCTCTTTTGAGGAACAAGTTTTCTTGTAGCGATAGCT**
69	**TAGATTAAGATCCTCTTTTGAGGAACAAGTTTTCTTGTCGCTGAGAAG**
70	**AGTCAATAGTTCCTCTTTTGAGGAACAAGTTTTCTTGTGAATTTATCA**
71	**AAATCATAGGTCCTCTTTTGAGGAACAAGTTTTCTTGTTCTGAGAGAC**
72	**TACCTTTTTATCCTCTTTTGAGGAACAAGTTTTCTTGTACCTCCGGCT**
73	**TAGGTTGGGTTCCTCTTTTGAGGAACAAGTTTTCTTGTTATATAACTA**
74	**TATGTAAATGTCCTCTTTTGAGGAACAAGTTTTCTTGTCTGATGCAAA**
75	**TCCAATCGCATCCTCTTTTGAGGAACAAGTTTTCTTGTAGACAAAGAA**
76	CGCGAGAAAACTTTTTCAAATATATTTTAGTTAATTTCATCTTCTG
77	ACCTAAATTTAATGGTTTGAAATACCGACCGTGTGATA
78	AATAAGGCGTTAAATAAGAATAAACACCGGAATCATAA

79	TTACTAGAAAAAGCCTGTTTAGTATCATATGCGTTATA
80	CAAATTCTTACCAGTATAAAGCCAACGCTCAACAGTAG
81	GGCTTAATTGAGAATCGCCATATTTAACAACGCCAACA
82	TGTAATTTAGGCAGAGGCATTTTCGAGCCAGTAATAAG
83	AGAATATAAAGTACCGACAAAAGGTAAAGTAATTCTGT
84	CCAGACGACGACAATAAACAACATGTTCAGCTAATGCA
85	GAACGCGCCTGTTTATCAACAATAGATAAGTCCTGAAC
86	AAGAAAAATAATATCCCATCCTAATTTACGAGCATGTA
87	GAAACCAATCAATAATCGGCTGTCTTTCCTTATCATTC
88	CAAGAACGGGTATTAAACCAAGTACCGCACTCATCGAG
89	AACAAGCAAGCCGTTTTTATTTTCATCGTAGGAATCAT
90	TACCGCGCCCAATAGCAAGCAAATCAGATATAGAAGGC
91	TTATCCGGTATTCTAAGAACGCGAGGCGTTTTAGCGAA
92	CCTCCCGACTTGCGGGAGGTTTTGAAGCCTTAAATCAA
93	GATTAGTTGCTATTTTGCACCCAGCTACAATTTTATCC
94	TGAATCTTACCAACGCTAACGAGCGTCTTTCCAGAGCC
95	TAATTTGCCAGTTACAAAATAAACAGCCATATTATTTA
96	TCCCAATCCAAATAAGAAACGATTTTTTGTTTAACGTC
97	AAAAATGAAAATAGCAGCCTTTACAGAGAGAATAACAT
98	AAAAACAGGGAAGCGCATTAGACGGGAGAATTAACTGA
99	ACACCCTGAACAAAGTCAGAGGGTAATTGAGCGCTAAT
100	GAATTGAGTTAAGCCCAA
101	**ATCAGAGAGATAACCCACAAttttTACGGATGTATAAGCATTCACTGATTCCG**
102	TAATAAGAGCAAGAAACAATGAAATAGCAATAGCTATC
103	TTACCGAAGCCCTTTTTAAGAAAAGTAAGCAGATAGCC
104	GAACAAAGTTACCAGAAGGAAACCGAGGAAACGCAATA
105	ATAACGGAATACCCAAAAGAACTGGCATGATTAAGACT
106	CCTTATTACGCAGTATGTTAGCAAACGTAGAAAATACA
107	TACATAAAGGTGGCAACATATAAAAGAAACGCAAAGAC
108	ACCACGGAATAAGTTTATTTTGTCACAATCAATAGAAA
109	ATTCATATGGTTTACCAGCGCCAAAGACAAAAGGGCGA
110	CATTCAACCGATTGAGGGAGGGAAGGTAAATATTGACG
111	GAAATTATTCATTAAAGGTGAATTATCACCGTCACCGA
112	CTTGAGCCATTTGGGAATTAGAGCCAGCAAAATCACCA
113	GTAGCACCATTACCATTAGCAAGGCCGGAAACGTCACC
114	AATGAAACCATCGATAGCAGCACCGTAATCAGTAGCGA
115	CAGAATCAAGTTTGCCTTTAGCGTCAGACTGTAGCGCG
116	TTTTCATCGGCATTTTCGGTCATAGCCCCCTTATTAGC
117	GTTTGCCATCTTTTCATAATCAAAATCACCGGAACCAG
118	AGCCACCACCGGAACCGCCTCCCTCAGAGCCGCCACCC
119	TCAGAACCGCCACCCTCAGAGCCACCACCCTCAGAGCC
120	GCCACCAGAACCACCACCAGAGCCGCCGCCAGCATTGA
121	CAGGAGGTTGAGGCAGGTCAGACGATTGGCCTTGATAT

122	TCACAAACAAATAAATCCTCATTAAAGCCAGAATGGAA
123	AGCGCAGTCTCTGAATTTACCGTTCCAGTAAGCGTCAT
124	ACATGGCTTTTGATGATACAGGAGTGTACTGGTAATAA
125	GTTTTAACGGGGTCAGTGCCTTGAGTAACAGTGCCCGT
126	ATAAACAGTTAATGCCCCCTGCCTATTTCGGAACCTAT
127	TATTCTGAAACATGAAAGTATTAAGAGGCTGAGACTCC
128	TCAAGAGAAGGATTAGGATTAGCGGGGTTTTGCTCAGT
129	ACCAGGCGGATAAGTGCCGTCGAGAGGGTTGATATAAG
130	TATAGCCCGGAATAGGTGTATCACCGTACTCAGGAGGT
131	TTAGTACCGCCACCCTCAGAACCGCCACCCTCAGAACC
132	GCCACCCTCAGAGCCACCACCCTCATTTTCAGGGATAG
133	CAAGCCCAATAGGAACCCATGTACCGTAACACTGAGTT
134	TCGTCACCAGTACAAACTACAACGCCTGTAGCATTCCA
135	CAGACAGCCCTCATAGTTAGCGTAACGATCTAAAGTTT
136	TGTCGTCTTTCCAGACGTTAGTAAATGAATTTTCTGTA
137	TGGGATTTTGCTAAACAACTTTCAACAGTTTCAGCGGA
138	GTGAGAATAGAAAGGAACAACTAAAGGAATTGCGAATA
139	ATAATTTTTTCACGTTGAAAATCTCCAAAAAAAAGGCT
140	CCAAAAGGAGCCTTTAATTGTATCGGTTTATCAGCTTG
141	CTTTCGAGGTGAATTTCTTAAACAGCTTGATACCGATA
142	GTTGCGCCGACAATGACAACAACCATCGCCCACGCATA
143	ACCGATATATTCGGTCGCTGAGGCTTGCAGGGAGTTAA
144	AGGCCGCTTTTGCGGGATCGTCACCCTCAGCAGCGAAA
145	GACAGCATCGGAACGAGGGTAGCAACGGCTACAGAGGC
146	TTTGAGGACTAAAGACTTTTTCATGAGGAAGTTTCCAT
147	TAAACGGGTAAAATACGTAATGCCACTACGAAGGCACC
148	AACCTAAAACGAAAGAGGCAAAAGAATACACTAAAACA
149	CTCATCTTTGACCCCCAGCGATTATACCAAGCGCGAAA
150	CAAAGTACAACGGAGATTTGTATCATCGCCTGATAAAT
151	TGTGTCGAAATCCGCGACCTGCTCCATGTTACTTAGCC
152	GGAACGAGGCGCAGACGGTCAATCATAAGGGAACCGAA
153	CTGACCAACTTTGAAAGAGGACAGATGAACGGTGTACA
154	GACCAGGCGCATAGGCTGGCTGACCTTCATCAAGAGTA
155	ATCTTGACAAGAACCGGATATTCATTACCCAAATCAAC
156	GTAACAAAGCTGCTCATTCAGTGAATAAGGCTTGCCCT
157	GACGAGAAACACCAGAACGAGTAGTAAATTGGGCTTGA
158	GATGGTTTAATTTCAACTTTAATCATTGTGAATTACCT
159	TATGCGATTTTAAGAACTGGCTCATTATACCAGTCAGG
160	ACGTTGGGAAGAAAAATCTACGTTAATAAAACGAACTA
161	ACGGAACAACATTATTACAGGTAGAAAGATTCATCAGT
162	TGAGATTTAGGAATACCACATTCAACTAATGCAGATAC
163	ATAACGCCAAAAGGAATTACGAGGCATAGTAAGAGCAA
164	CACTATCATAACCCTCGTTTACCAGACGACGATAAAAA

165	CCAAAATAGCGAGAGGCTTTTGCAAAAGAAGTTTTGCC
166	AGAGGGGGTAATAGTAAAATGTTTAGACTGGATAGCGT
167	CCAATACTGCGGAATCGTCATAAATATTCATTGAATCC
168	CCCTCAAATGCTTTAAACAGTTCAGAAAACGAGAATGA
169	CCATAAATCAAAAATCAGGTCTTTACCCTGACTATTAT
170	AGTCAGAAGCAAAGCGGATTGCATCAAAAAGATTAAGA
171	GGAAGCCCGAAAGACTTCAAATATCGCGTTTTAATTCG
172	AGCTTCAAAGCGAACCAGACCGGAAGCAAACTCCAACA
173	GGTCAGGATTAGAGAGTACCTTTAATTGCTCCTTTTGA
174	TAAGAGGTCATTTTTGCGGATGGCTTAGAGCTTAATTG
175	CTGAATATAATGCTGTAGCTCAACATGTTTTAAATATG
176	CAACTAAAGTACGGTGTCTGGAAGTTTCATTCCATATA
177	ACAGTTGATTCCCAATTCTGCGAACGAGTAGATTTAGT
178	TTGACCATTAGATACATTTCGCAAATGGTCAATAACCT
179	GTTTAGCTATATTTTCATTTGGGGCGCGAGCTGAAAAG
180	GTGGCATCAATTCTACTAATAGTAGTAGCATTAACATC
181	CAATAAATCATACAGGCAAGGCAAAGAATTAGCAAAAT
182	TAAGCAATAAAGCCTCAGAGCATAAAGCTAAATCGGTT
183	GTACCAAAAACATTATGACCCTGTAATACTTTTGCGGG
184	AGAAGCCTTTATTTCAACGCAAGGATAAAAATTTTTAG
185	AACCCTCATATATTTTAAATGCAATGCCTGAGTAATGT
186	GTAGGTAAAGATTCAAAAGGGTGAGAAAGGCCGGAGAC
187	AGTCAAATCACCATCAATATGATATTCAACCGTTCTAG
188	CTGATAAATTAATGCCGGAGAGGGTAGCTATTTTTGAG
189	AGATCTACAAAGGCTATCAGGTCATTGCCTGAGAGTCT
190	GGAGCAAACAAGAGAATCGATGAACGGTAATCGTAAAA
191	CTAGCATGTCAATCATATGTACCCCGGTTGATAATCAG
192	AAAAGCCCCAAAAACAGGAAGATTGTATAAGCAAATAT
193	TTAAATTGTAAACGTTAATATTTTGTTAAAATTCGCAT
194	TAAATTTTTGTTAAATCAGCTCATTTTTTAACCAATAG
195	GAACGCCATCAAAAATAATTCGCGTCTGGCCTTCCTGT
196	AGCCAGCTTTCATCAACATTAAATGTGAGCGAGTAACA
197	ACCCGTCGGATTCTCCGTGGGAACAAACGGCGGATTGA
198	CCGTAATGGGATAGGTCACGTTGGTGTAGATGGGCGCA
199	TCGTAACCGTGCATCTGCCAGTTTGAGGGGACGACGAC
200	AGTATCGGCCTCAGGAAGATCGCACTCCAGCCAGCTTT
201	CCGGCACCGCTTCTGGTGCCGGAAACCAGGCAAAGCGC
202	CATTCGCCATTCAGGCTGCGCAACTGTTGGGAAGGGCG
203	ATCGGTGCGGGCCTCTTCGCTATTACGCCAGCTGGCGA
204	AAGGGGGATGTGCTGCAAGGCGATTAAGTTGGGTAACG
205	CCAGGGTTTTCCCAGTCACGACGTTGTAAAACGACGGC
206	CAGTGCCAAGCTTGCATGCCTGCAGGTCGACTCTAGAGGATCTTTT

Table S4.

Sequence of DNA oligonucleotides used for assembly of dsDNA nanostructure used for capture of non-PEGylated pegaptanib 'R'.

Oligonucleotide / **Dumbbell** / **Overhang**

Oligo number	Sequence (5′→ 3′)
1	TTTTCGTAATCATGGTCATAGCTGTTTCCTGTGTGAAATTGTTATC
2	CGCTCACAATTCCACACAACATACGAGCCGGAAGCATA
3	AAGTGTAAAGCCTGGGGTGCCTAATGAGTGAGCTAACT
4	CACATTAATTGCGTTGCGCTCACTGCCCGCTTTCCAGT
5	CGGGAAACCTGTCGTGCCAGCTGCATTAATGAATCGGC
6	CAACGCGCGGGGAGAGGCGGTTTGCGTATTGGGCGCCA
7	GGGTGGTTTTTCTTTTCACCAGTGAGACGGGCAACAGC
8	TGATTGCCCTTCACCGCCTGGCCCTGAGAGAGTTGCAG
9	CAAGCGGTCCACGCTGGTTTGCCCCAGCAGGCGAAAAT
10	CCTGTTTGATGGTGGTTCCGAAATCGGCAAAATCCCTT
11	ATAAATCAAAAGAATAGCCCGAGATAGGGTTGAGTGTT
12	GTTCCAGTTTGGAACAAGAGTCCACTATTAAAGAACGT
13	GGACTCCAACGTCAAAGGGCGAAAAACCGTCTATCAGG
14	GCGATGGCCCACTACGTGAACCATCACCCAAATCAAGT
15	TTTTTGGGGTCGAGGTGCCGTAAAGCACTAAATCGGAA
16	CCCTAAAGGGAGCCCCCGATTTAGAGCTTGACGGGGAA
17	AGCCGGCGAACGTGGCGAGAAAGGAAGGGAAGAAAGCG
18	AAAGGAGCGGGCGCTAGGGCGCTGGCAAGTGTAGCGGT
19	CACGCTGCGCGTAACCACCACACCCGCCGCGCTTAATG
20	CGCCGCTACAGGGCGCGTACTATGGTTGCTTTGACGAG
21	CACGTATAACGTGCTTTCCTCGTTAGAATCAGAGCGGG
22	AGCTAAACAGGAGGCCGATTAAAGGGATTTTAGACAGG
23	AACGGTACGCCAGAATCCTGAGAAGTGTTTTTATAATC
24	AGTGAGGCCACCGAGTAAAAGAGTCTGTCCATCACGCA
25	AATTAACCGTTGTAGCAATACTTCTTTGATTAGTAATA
26	**ACATCACTTGTCCTCTTTTGAGGAACAAGTTTTCTTGTCCTGAGTAGA**
27	**AGAACTCAAATCCTCTTTTGAGGAACAAGTTTTCTTGTCTATCGGCCT**
28	**TGCTGGTAATTCCTCTTTTGAGGAACAAGTTTTCTTGTATCCAGAACA**
29	**ATATTACCGCTCCTCTTTTGAGGAACAAGTTTTCTTGTCAGCCATTGC**
30	**AACAGGAAAATCCTCTTTTGAGGAACAAGTTTTCTTGTACGCTCATGG**
31	**AAATACCTACTCCTCTTTTGAGGAACAAGTTTTCTTGTATTTTGACGC**
32	**TCAATCGTCTTCCTCTTTTGAGGAACAAGTTTTCTTGTGAAATGGATT**

33	**ATTTACATTGTCCTCTTTTGAGGAACAAGTTTTCTTGTGCAGATTCAC**
34	**CAGTCACACGTCCTCTTTTGAGGAACAAGTTTTCTTGTACCAGTAATA**
35	**AAAGGGACATTCCTCTTTTGAGGAACAAGTTTTCTTGTTCTGGCCAAC**
36	**AGAGATAGAATCCTCTTTTGAGGAACAAGTTTTCTTGTCCCTTCTGAC**
37	CTGAAAGCGTAAGAATACGTGGCACAGACAATATTTTTGAATGGCT
38	ATTAGTCTTTAATGCGCGAACTGATAGCCCTAAAACAT
39	CGCCATTAAAAATACCGAACGAACCACCAGCAGAAGAT
40	AAAACAGAGGTGAGGCGGTCAGTATTAACACCGCCTGC
41	AACAGTGCCACGCTGAGAGCCAGCAGCAAATGAAAAAT
42	CTAAAGCATCACCTTGCTGAACCTCAAATATCAAACCC
43	TCAATCAATATCTGGTCAGTTGGCAAATCAACAGTTGA
44	AAGGAATTGAGGAAGGTTATCTAAAATATCTTTAGGAG
45	AATATAATCCTGATTGTTTGGATTATACTTCTGAATAATGGAAGGG
46	**CACTAACAACTCCTCTTTTGAGGAACAAGTTTTCTTGTTAATAGATTA**
47	**GAGCCGTCAATCCTCTTTTGAGGAACAAGTTTTCTTGTTAGATAATAC**
48	**ATTTGAGGATTCCTCTTTTGAGGAACAAGTTTTCTTGTTTAGAAGTAT**
49	**TAGACTTTACTCCTCTTTTGAGGAACAAGTTTTCTTGTAAACAATTCG**
50	**ACAACTCGTATCCTCTTTTGAGGAACAAGTTTTCTTGTTTAAATCCTT**
51	**TGCCCGAACGTCCTCTTTTGAGGAACAAGTTTTCTTGTTTATTAATTT**
52	**TAAAAGTTTGTCCTCTTTTGAGGAACAAGTTTTCTTGTAGTAACATTA**
53	**TCATTTTGCGTCCTCTTTTGAGGAACAAGTTTTCTTGTGAACAAAGAA**
54	**ACCACCAGAATCCTCTTTTGAGGAACAAGTTTTCTTGTGGAGCGGAAT**
55	**TATCATCATATCCTCTTTTGAGGAACAAGTTTTCTTGTTTCCTGATTA**
56	**TCAGATGATGTCCTCTTTTGAGGAACAAGTTTTCTTGTGCAATTCATC**
57	TTAGAACCTACCATATCAAAATTATTTGCACGTAAAAC
58	AGAAATAAAGAAATTGCGTAGATTTTCAGGTTTAACGT
59	CAGATGAATATACAGTAACAGTACCTTTTACATCGGGA
60	GAAACAATAACGGATTCGCCTGATTGCTTTGAATACCA
61	AGTTACAAAATCGCGCAGAGGCGAATTATTCATTTCAA
62	TTACCTGAGCAAAAGAAGATGATGAAACAAACATCAAG
63	AAAACAAAATTAATTACATTTAACAATTTCATTTGAAT
64	TACCTTTTTTAATGGAAACAGTACATAAATCAATATAT
65	GTGAGTGAATAACCTTGCTTCTGTAAATCGTCGCTATT
66	AATTAATTTTCCCTTAGAATCCTTGAAAACATAGCGAT
67	AGCTTAGATTAAGACGCTGAGAAGAGTCAATAGTGAAT
68	TTATCAAAATCATAGGTCTGAGAGACTACCTTTTTAAC
69	CTCCGGCTTAGGTTGGGTTATATAACTATATGTAAATG
70	CTGATGCAAATCCAATCGCAAGACAAAGAACGCGAGAA
71	AACTTTTTCAAATATATTTTAGTTAATTTCATCTTCTG
72	ACCTAAATTTAATGGTTTGAAATACCGACCGTGTGATA
73	AATAAGGCGTTAAATAAGAATAAACACCGGAATCATAA
74	TTACTAGAAAAAGCCTGTTTAGTATCATATGCGTTATA
75	CAAATTCTTACCAGTATAAAGCCAACGCTCAACAGTAG

76	GGCTTAATTGAGAATCGCCATATTTAACAACGCCAACA
77	TGTAATTTAGGCAGAGGCATTTTCGAGCCAGTAATAAG
78	AGAATATAAAGTACCGACAAAAGGTAAAGTAATTCTGT
79	CCAGACGACGACAATAAACAACATGTTCAGCTAATGCA
80	GAACGCGCCTGTTTATCAACAATAGATAAGTCCTGAAC
81	AAGAAAAATAATATCCCATCCTAATTTACGAGCATGTA
82	GAAACCAATCAATAATCGGCTGTCTTTCCTTATCATTC
83	CAAGAACGGGTATTAAACCAAGTACCGCACTCATCGAG
84	AACAAGCAAGCCGTTTTTATTTTCATCGTAGGAATCAT
85	TACCGCGCCCAATAGCAAGCAAATCAGATATAGAAGGC
86	TTATCCGGTATTCTAAGAACGCGAGGCGTTTTAGCGAA
87	CCTCCCGACTTGCGGGAGGTTTTGAAGCCTTAAATCAA
88	GATTAGTTGCTATTTTGCACCCAGCTACAATTTTATCC
89	TGAATCTTACCAACGCTAACGAGCGTCTTTCCAGAGCC
90	TAATTTGCCAGTTACAAAATAAACAGCCATATTATTTA
91	TCCCAATCCAAATAAGAAACGATTTTTTGTTTAACGTC
92	AAAAATGAAAATAGCAGCCTTTACAGAGAGAATAACAT
93	AAAAACAGGGAAGCGCATTAGACGGGAGAATTAACTGA
94	ACACCCTGAACAAAGTCAGAGGGTAATTGAGCGCTAAT
95	GAATTGAGTTAAGCCCAA
96	**ATCAGAGAGATAACCCACAAttttTACGGATGTATAAGCATTCACTGATTCCG**
97	TAATAAGAGCAAGAAACAATGAAATAGCAATAGCTATC
98	TTACCGAAGCCCTTTTTAAGAAAAGTAAGCAGATAGCC
99	GAACAAAGTTACCAGAAGGAAACCGAGGAAACGCAATA
100	ATAACGGAATACCCAAAAGAACTGGCATGATTAAGACT
101	CCTTATTACGCAGTATGTTAGCAAACGTAGAAAATACA
102	TACATAAAGGTGGCAACATATAAAAGAAACGCAAAGAC
103	ACCACGGAATAAGTTTATTTTGTCACAATCAATAGAAA
104	ATTCATATGGTTTACCAGCGCCAAAGACAAAAGGGCGA
105	CATTCAACCGATTGAGGGAGGGAAGGTAAATATTGACG
106	GAAATTATTCATTAAAGGTGAATTATCACCGTCACCGA
107	CTTGAGCCATTTGGGAATTAGAGCCAGCAAAATCACCA
108	GTAGCACCATTACCATTAGCAAGGCCGGAAACGTCACC
109	AATGAAACCATCGATAGCAGCACCGTAATCAGTAGCGA
110	CAGAATCAAGTTTGCCTTTAGCGTCAGACTGTAGCGCG
111	TTTTCATCGGCATTTTCGGTCATAGCCCCCTTATTAGC
112	GTTTGCCATCTTTTCATAATCAAAATCACCGGAACCAG
113	AGCCACCACCGGAACCGCCTCCCTCAGAGCCGCCACCC
114	TCAGAACCGCCACCCTCAGAGCCACCACCCTCAGAGCC
115	GCCACCAGAACCACCACCAGAGCCGCCGCCAGCATTGA
116	CAGGAGGTTGAGGCAGGTCAGACGATTGGCCTTGATAT
117	TCACAAACAAATAAATCCTCATTAAAGCCAGAATGGAA
118	AGCGCAGTCTCTGAATTTACCGTTCCAGTAAGCGTCAT

119	ACATGGCTTTTGATGATACAGGAGTGTACTGGTAATAA
120	GTTTTAACGGGGTCAGTGCCTTGAGTAACAGTGCCCGT
121	ATAAACAGTTAATGCCCCCTGCCTATTTCGGAACCTAT
122	TATTCTGAAACATGAAAGTATTAAGAGGCTGAGACTCC
123	TCAAGAGAAGGATTAGGATTAGCGGGGTTTTGCTCAGT
124	ACCAGGCGGATAAGTGCCGTCGAGAGGGTTGATATAAG
125	TATAGCCCGGAATAGGTGTATCACCGTACTCAGGAGGT
126	TTAGTACCGCCACCCTCAGAACCGCCACCCTCAGAACC
127	GCCACCCTCAGAGCCACCACCCTCATTTTCAGGGATAG
128	CAAGCCCAATAGGAACCCATGTACCGTAACACTGAGTT
129	TCGTCACCAGTACAAACTACAACGCCTGTAGCATTCCA
130	CAGACAGCCCTCATAGTTAGCGTAACGATCTAAAGTTT
131	TGTCGTCTTTCCAGACGTTAGTAAATGAATTTTCTGTA
132	TGGGATTTTGCTAAACAACTTTCAACAGTTTCAGCGGA
133	GTGAGAATAGAAAGGAACAACTAAAGGAATTGCGAATA
134	ATAATTTTTTCACGTTGAAAATCTCCAAAAAAAAGGCT
135	CCAAAAGGAGCCTTTAATTGTATCGGTTTATCAGCTTG
136	CTTTCGAGGTGAATTTCTTAAACAGCTTGATACCGATA
137	GTTGCGCCGACAATGACAACAACCATCGCCCACGCATA
138	ACCGATATATTCGGTCGCTGAGGCTTGCAGGGAGTTAA
139	AGGCCGCTTTTGCGGGATCGTCACCCTCAGCAGCGAAA
140	GACAGCATCGGAACGAGGGTAGCAACGGCTACAGAGGC
141	TTTGAGGACTAAAGACTTTTTCATGAGGAAGTTTCCAT
142	TAAACGGGTAAAATACGTAATGCCACTACGAAGGCACC
143	AACCTAAAACGAAAGAGGCAAAAGAATACACTAAAACA
144	CTCATCTTTGACCCCCAGCGATTATACCAAGCGCGAAA
145	CAAAGTACAACGGAGATTTGTATCATCGCCTGATAAAT
146	TGTGTCGAAATCCGCGACCTGCTCCATGTTACTTAGCC
147	GGAACGAGGCGCAGACGGTCAATCATAAGGGAACCGAA
148	CTGACCAACTTTGAAAGAGGACAGATGAACGGTGTACA
149	GACCAGGCGCATAGGCTGGCTGACCTTCATCAAGAGTA
150	ATCTTGACAAGAACCGGATATTCATTACCCAAATCAAC
151	GTAACAAAGCTGCTCATTCAGTGAATAAGGCTTGCCCT
152	GACGAGAAACACCAGAACGAGTAGTAAATTGGGCTTGA
153	GATGGTTTAATTTCAACTTTAATCATTGTGAATTACCT
154	TATGCGATTTTAAGAACTGGCTCATTATACCAGTCAGG
155	ACGTTGGGAAGAAAAATCTACGTTAATAAAACGAACTA
156	ACGGAACAACATTATTACAGGTAGAAAGATTCATCAGT
157	TGAGATTTAGGAATACCACATTCAACTAATGCAGATAC
158	ATAACGCCAAAAGGAATTACGAGGCATAGTAAGAGCAA
159	CACTATCATAACCCTCGTTTACCAGACGACGATAAAAA
160	CCAAAATAGCGAGAGGCTTTTGCAAAAGAAGTTTTGCC
161	AGAGGGGGTAATAGTAAAATGTTTAGACTGGATAGCGT

162	CCAATACTGCGGAATCGTCATAAATATTCATTGAATCC
163	CCCTCAAATGCTTTAAACAGTTCAGAAAACGAGAATGA
164	CCATAAATCAAAAATCAGGTCTTTACCCTGACTATTAT
165	AGTCAGAAGCAAAGCGGATTGCATCAAAAAGATTAAGA
166	**GGAAGCCCGATCCTCTTTTGAGGAACAAGTTTTCTTGTAAGACTTCAA**
167	**ATATCGCGTTTCCTCTTTTGAGGAACAAGTTTTCTTGTTTAATTCGAG**
168	**CTTCAAAGCGTCCTCTTTTGAGGAACAAGTTTTCTTGTAACCAGACCG**
169	**GAAGCAAACTTCCTCTTTTGAGGAACAAGTTTTCTTGTCCAACAGGTC**
170	**AGGATTAGAGTCCTCTTTTGAGGAACAAGTTTTCTTGTAGTACCTTTA**
171	**ATTGCTCCTTTCCTCTTTTGAGGAACAAGTTTTCTTGTTTGATAAGAG**
172	**GTCATTTTTGTCCTCTTTTGAGGAACAAGTTTTCTTGTCGGATGGCTT**
173	**AGAGCTTAATTCCTCTTTTGAGGAACAAGTTTTCTTGTTGCTGAATAT**
174	**AATGCTGTAGTCCTCTTTTGAGGAACAAGTTTTCTTGTCTCAACATGT**
175	**TTTAAATATGTCCTCTTTTGAGGAACAAGTTTTCTTGTCAACTAAAGT**
176	**ACGGTGTCTGTCCTCTTTTGAGGAACAAGTTTTCTTGTGAAGTTTCAT**
177	**TCCATATAACTCCTCTTTTGAGGAACAAGTTTTCTTGTAGTTGATTCC**
178	CAATTCTGCGAACGAGTAGATTTAGT
179	TTGACCATTAGATACATTTCGCAAATGGTCAATAACCT
180	GTTTAGCTATATTTTCATTTGGGGCGCGAGCTGAAAAG
181	GTGGCATCAATTCTACTAATAGTAGTAGCATTAACATC
182	CAATAAATCATACAGGCAAGGCAAAGAATTAGCAAAAT
183	TAAGCAATAAAGCCTCAGAGCATAAAGCTAAATCGGTT
184	GTACCAAAAACATTATGACCCTGTAATACTTTTGCGGG
185	AGAAGCCTTTATTTCAACGCAAGGATAAAAATTTTTAG
186	AACCCTCATATATTTTAAATGCAATGCCTGAGTAATGT
187	GTAGGTAAAGATTCAAAAGGGTGAGAAAGGCCGGAGAC
188	AGTCAAATCACCATCAATATGATATTCAACCGTTCTAG
189	CTGATAAATTAATGCCGGAGAGGGTAGCTATTTTTGAG
190	AGATCTACAAAGGCTATCAGGTCATTGCCTGAGAGTCT
191	GGAGCAAACAAGAGAATCGATGAACGGTAATCGTAAAA
192	CTAGCATGTCAATCATATGTACCCCGGTTGATAATCAG
193	AAAAGCCCCAAAAACAGGAAGATTGTATAAGCAAATAT
194	TTAAATTGTAAACGTTAATATTTTGTTAAAATTCGCAT
195	TAAATTTTTGTTAAATCAGCTCATTTTTTAACCAATAG
196	GAACGCCATCAAAAATAATTCGCGTCTGGCCTTCCTGT
197	AGCCAGCTTTCATCAACATTAAATGTGAGCGAGTAACA
198	ACCCGTCGGATTCTCCGTGGGAACAAACGGCGGATTGA
199	CCGTAATGGGATAGGTCACGTTGGTGTAGATGGGCGCA
200	TCGTAACCGTGCATCTGCCAGTTTGAGGGGACGACGAC
201	AGTATCGGCCTCAGGAAGATCGCACTCCAGCCAGCTTT
202	CCGGCACCGCTTCTGGTGCCGGAAACCAGGCAAAGCGC
203	CATTCGCCATTCAGGCTGCGCAACTGTTGGGAAGGGCG
204	ATCGGTGCGGGCCTCTTCGCTATTACGCCAGCTGGCGA

205	AAGGGGGATGTGCTGCAAGGCGATTAAGTTGGGTAACG
206	CCAGGGTTTTCCCAGTCACGACGTTGTAAAACGACGGC
207	CAGTGCCAAGCTTGCATGCCTGCAGGTCGACTCTAGAGGATCTTTT

Table S5.

Sequence of 1) biotinylated RNA aptamer.

**Oligo number**	**Sequence (5′→ 3′)**
**1**	3′-modification CGGAAUCAGUGAAUGCUUAUACAUCCGU-biotin

Sequence of DNA oligonucleotides used for assembly of dsDNA nanostructure used for capture of biotinylated aptamer ‘S’.

Oligonucleotide / **Dumbbell** / **Overhang**

**Oligo number**	**Sequence (5′→ 3′)**
1	TTTTCGTAATCATGGTCATAGCTGTTTCCTGTGTGAAATTGTTATC
2	CGCTCACAATTCCACACAACATACGAGCCGGAAGCATA
3	AAGTGTAAAGCCTGGGGTGCCTAATGAGTGAGCTAACT
4	CACATTAATTGCGTTGCGCTCACTGCCCGCTTTCCAGT
5	CGGGAAACCTGTCGTGCCAGCTGCATTAATGAATCGGC
6	CAACGCGCGGGGAGAGGCGGTTTGCGTATTGGGCGCCA
7	GGGTGGTTTTTCTTTTCACCAGTGAGACGGGCAACAGC
8	TGATTGCCCTTCACCGCCTGGCCCTGAGAGAGTTGCAG
9	CAAGCGGTCCACGCTGGTTTGCCCCAGCAGGCGAAAAT
10	CCTGTTTGATGGTGGTTCCGAAATCGGCAAAATCCCTT
11	ATAAATCAAAAGAATAGCCCGAGATAGGGTTGAGTGTT
12	GTTCCAGTTTGGAACAAGAGTCCACTATTAAAGAACGT
13	GGACTCCAACGTCAAAGGGCGAAAAACCGTCTATCAGG
14	GCGATGGCCCACTACGTGAACCATCACCCAAATCAAGT
15	TTTTTGGGGTCGAGGTGCCGTAAAGCACTAAATCGGAA
16	CCCTAAAGGGAGCCCCCGATTTAGAGCTTGACGGGGAA
17	AGCCGGCGAACGTGGCGAGAAAGGAAGGGAAGAAAGCG
18	AAAGGAGCGGGCGCTAGGGCGCTGGCAAGTGTAGCGGT
19	CACGCTGCGCGTAACCACCACACCCGCCGCGCTTAATG
20	CGCCGCTACAGGGCGCGTACTATGGTTGCTTTGACGAG
21	CACGTATAACGTGCTTTCCTCGTTAGAATCAGAGCGGG
22	AGCTAAACAGGAGGCCGATTAAAGGGATTTTAGACAGG
23	AACGGTACGCCAGAATCCTGAGAAGTGTTTTTATAATC
24	AGTGAGGCCACCGAGTAAAAGAGTCTGTCCATCACGCA
25	AATTAACCGTTGTAGCAATACTTCTTTGATTAGTAATA
26	**ACATCACTTGTCCTCTTTTGAGGAACAAGTTTTCTTGTCCTGAGTAGA**

27	**AGAACTCAAATCCTCTTTTGAGGAACAAGTTTTCTTGTCTATCGGCCT**
28	**TGCTGGTAATTCCTCTTTTGAGGAACAAGTTTTCTTGTATCCAGAACA**
29	**ATATTACCGCTCCTCTTTTGAGGAACAAGTTTTCTTGTCAGCCATTGC**
30	**AACAGGAAAATCCTCTTTTGAGGAACAAGTTTTCTTGTACGCTCATGG**
31	**AAATACCTACTCCTCTTTTGAGGAACAAGTTTTCTTGTATTTTGACGC**
32	**TCAATCGTCTTCCTCTTTTGAGGAACAAGTTTTCTTGTGAAATGGATT**
33	**ATTTACATTGTCCTCTTTTGAGGAACAAGTTTTCTTGTGCAGATTCAC**
34	**CAGTCACACGTCCTCTTTTGAGGAACAAGTTTTCTTGTACCAGTAATA**
35	**AAAGGGACATTCCTCTTTTGAGGAACAAGTTTTCTTGTTCTGGCCAAC**
36	**AGAGATAGAATCCTCTTTTGAGGAACAAGTTTTCTTGTCCCTTCTGAC**
37	CTGAAAGCGTAAGAATACGTGGCACAGACAATATTTTTGAATGGCT
38	ATTAGTCTTTAATGCGCGAACTGATAGCCCTAAAACAT
39	CGCCATTAAAAATACCGAACGAACCACCAGCAGAAGAT
40	AAAACAGAGGTGAGGCGGTCAGTATTAACACCGCCTGC
41	AACAGTGCCACGCTGAGAGCCAGCAGCAAATGAAAAAT
42	CTAAAGCATCACCTTGCTGAACCTCAAATATCAAACCC
43	TCAATCAATATCTGGTCAGTTGGCAAATCAACAGTTGA
44	AAGGAATTGAGGAAGGTTATCTAAAATATCTTTAGGAG
45	AATATAATCCTGATTGTTTGGATTATACTTCTGAATAATGGAAGGG
46	**CACTAACAACTCCTCTTTTGAGGAACAAGTTTTCTTGTTAATAGATTA**
47	**GAGCCGTCAATCCTCTTTTGAGGAACAAGTTTTCTTGTTAGATAATAC**
48	**ATTTGAGGATTCCTCTTTTGAGGAACAAGTTTTCTTGTTTAGAAGTAT**
49	**TAGACTTTACTCCTCTTTTGAGGAACAAGTTTTCTTGTAAACAATTCG**
50	**ACAACTCGTATCCTCTTTTGAGGAACAAGTTTTCTTGTTTAAATCCTT**
51	**TGCCCGAACGTCCTCTTTTGAGGAACAAGTTTTCTTGTTTATTAATTT**
52	**TAAAAGTTTGTCCTCTTTTGAGGAACAAGTTTTCTTGTAGTAACATTA**
53	**TCATTTTGCGTCCTCTTTTGAGGAACAAGTTTTCTTGTGAACAAAGAA**
54	**ACCACCAGAATCCTCTTTTGAGGAACAAGTTTTCTTGTGGAGCGGAAT**
55	**TATCATCATATCCTCTTTTGAGGAACAAGTTTTCTTGTTTCCTGATTA**
56	**TCAGATGATGTCCTCTTTTGAGGAACAAGTTTTCTTGTGCAATTCATC**
57	TTAGAACCTACCATATCAAAATTATTTGCACGTAAAAC
58	AGAAATAAAGAAATTGCGTAGATTTTCAGGTTTAACGT
59	CAGATGAATATACAGTAACAGTACCTTTTACATCGGGA
60	GAAACAATAACGGATTCGCCTGATTGCTTTGAATACCA
61	AGTTACAAAATCGCGCAGAGGCGAATTATTCATTTCAA
62	TTACCTGAGCAAAAGAAGATGATGAAACAAACATCAAG
63	AAAACAAAATTAATTACATTTAACAATTTCATTTGAAT
64	TACCTTTTTTAATGGAAACAGTACATAAATCAATATAT
65	GTGAGTGAATAACCTTGCTTCTGTAAATCGTCGCTATT
66	AATTAATTTTCCCTTAGAATCCTTGAAAACATAGCGAT
67	AGCTTAGATTAAGACGCTGAGAAGAGTCAATAGTGAAT
68	TTATCAAAATCATAGGTCTGAGAGACTACCTTTTTAAC
69	CTCCGGCTTAGGTTGGGTTATATAACTATATGTAAATG

70	CTGATGCAAATCCAATCGCAAGACAAAGAACGCGAGAA
71	AACTTTTTCAAATATATTTTAGTTAATTTCATCTTCTG
72	ACCTAAATTTAATGGTTTGAAATACCGACCGTGTGATA
73	AATAAGGCGTTAAATAAGAATAAACACCGGAATCATAA
74	TTACTAGAAAAAGCCTGTTTAGTATCATATGCGTTATA
75	CAAATTCTTACCAGTATAAAGCCAACGCTCAACAGTAG
76	GGCTTAATTGAGAATCGCCATATTTAACAACGCCAACA
77	TGTAATTTAGGCAGAGGCATTTTCGAGCCAGTAATAAG
78	AGAATATAAAGTACCGACAAAAGGTAAAGTAATTCTGT
79	CCAGACGACGACAATAAACAACATGTTCAGCTAATGCA
80	GAACGCGCCTGTTTATCAACAATAGATAAGTCCTGAAC
81	AAGAAAAATAATATCCCATCCTAATTTACGAGCATGTA
82	GAAACCAATCAATAATCGGCTGTCTTTCCTTATCATTC
83	CAAGAACGGGTATTAAACCAAGTACCGCACTCATCGAG
84	AACAAGCAAGCCGTTTTTATTTTCATCGTAGGAATCAT
85	TACCGCGCCCAATAGCAAGCAAATCAGATATAGAAGGC
86	TTATCCGGTATTCTAAGAACGCGAGGCGTTTTAGCGAA
87	CCTCCCGACTTGCGGGAGGTTTTGAAGCCTTAAATCAA
88	GATTAGTTGCTATTTTGCACCCAGCTACAATTTTATCC
89	TGAATCTTACCAACGCTAACGAGCGTCTTTCCAGAGCC
90	TAATTTGCCAGTTACAAAATAAACAGCCATATTATTTA
91	TCCCAATCCAAATAAGAAACGATTTTTTGTTTAACGTC
92	AAAAATGAAAATAGCAGCCTTTACAGAGAGAATAACAT
93	AAAAACAGGGAAGCGCATTAGACGGGAGAATTAACTGA
94	ACACCCTGAACAAAGTCAGAGGGTAATTGAGCGCTAAT
95	GAATTGAGTTAAGCCCAA
96	**ACGGATGTATAAGCATTCACTGATTCCGTTTTTATCAGAGAGATAACCCACAA**
97	TAATAAGAGCAAGAAACAATGAAATAGCAATAGCTATC
98	TTACCGAAGCCCTTTTTAAGAAAAGTAAGCAGATAGCC
99	GAACAAAGTTACCAGAAGGAAACCGAGGAAACGCAATA
100	ATAACGGAATACCCAAAAGAACTGGCATGATTAAGACT
101	CCTTATTACGCAGTATGTTAGCAAACGTAGAAAATACA
102	TACATAAAGGTGGCAACATATAAAAGAAACGCAAAGAC
103	ACCACGGAATAAGTTTATTTTGTCACAATCAATAGAAA
104	ATTCATATGGTTTACCAGCGCCAAAGACAAAAGGGCGA
105	CATTCAACCGATTGAGGGAGGGAAGGTAAATATTGACG
106	GAAATTATTCATTAAAGGTGAATTATCACCGTCACCGA
107	CTTGAGCCATTTGGGAATTAGAGCCAGCAAAATCACCA
108	GTAGCACCATTACCATTAGCAAGGCCGGAAACGTCACC
109	AATGAAACCATCGATAGCAGCACCGTAATCAGTAGCGA
110	CAGAATCAAGTTTGCCTTTAGCGTCAGACTGTAGCGCG
111	TTTTCATCGGCATTTTCGGTCATAGCCCCCTTATTAGC
112	GTTTGCCATCTTTTCATAATCAAAATCACCGGAACCAG

113	AGCCACCACCGGAACCGCCTCCCTCAGAGCCGCCACCC
114	TCAGAACCGCCACCCTCAGAGCCACCACCCTCAGAGCC
115	GCCACCAGAACCACCACCAGAGCCGCCGCCAGCATTGA
116	CAGGAGGTTGAGGCAGGTCAGACGATTGGCCTTGATAT
117	TCACAAACAAATAAATCCTCATTAAAGCCAGAATGGAA
118	AGCGCAGTCTCTGAATTTACCGTTCCAGTAAGCGTCAT
119	ACATGGCTTTTGATGATACAGGAGTGTACTGGTAATAA
120	GTTTTAACGGGGTCAGTGCCTTGAGTAACAGTGCCCGT
121	ATAAACAGTTAATGCCCCCTGCCTATTTCGGAACCTAT
122	TATTCTGAAACATGAAAGTATTAAGAGGCTGAGACTCC
123	TCAAGAGAAGGATTAGGATTAGCGGGGTTTTGCTCAGT
124	ACCAGGCGGATAAGTGCCGTCGAGAGGGTTGATATAAG
125	TATAGCCCGGAATAGGTGTATCACCGTACTCAGGAGGT
126	TTAGTACCGCCACCCTCAGAACCGCCACCCTCAGAACC
127	GCCACCCTCAGAGCCACCACCCTCATTTTCAGGGATAG
128	CAAGCCCAATAGGAACCCATGTACCGTAACACTGAGTT
129	TCGTCACCAGTACAAACTACAACGCCTGTAGCATTCCA
130	CAGACAGCCCTCATAGTTAGCGTAACGATCTAAAGTTT
131	TGTCGTCTTTCCAGACGTTAGTAAATGAATTTTCTGTA
132	TGGGATTTTGCTAAACAACTTTCAACAGTTTCAGCGGA
133	GTGAGAATAGAAAGGAACAACTAAAGGAATTGCGAATA
134	ATAATTTTTTCACGTTGAAAATCTCCAAAAAAAAAGGCT
135	CCAAAAGGAGCCTTTAATTGTATCGGTTTATCAGCTTG
136	CTTTCGAGGTGAATTTCTTAAACAGCTTGATACCGATA
137	GTTGCGCCGACAATGACAACAACCATCGCCCACGCATA
138	ACCGATATATTCGGTCGCTGAGGCTTGCAGGGAGTTAA
139	AGGCCGCTTTTGCGGGATCGTCACCCTCAGCAGCGAAA
140	GACAGCATCGGAACGAGGGTAGCAACGGCTACAGAGGC
141	TTTGAGGACTAAAGACTTTTTCATGAGGAAGTTTCCAT
142	TAAACGGGTAAAATACGTAATGCCACTACGAAGGCACC
143	AACCTAAAACGAAAGAGGCAAAAGAATACACTAAAACA
144	CTCATCTTTGACCCCCAGCGATTATACCAAGCGCGAAA
145	CAAAGTACAACGGAGATTTGTATCATCGCCTGATAAAT
146	TGTGTCGAAATCCGCGACCTGCTCCATGTTACTTAGCC
147	GGAACGAGGCGCAGACGGTCAATCATAAGGGAACCGAA
148	CTGACCAACTTTGAAAGAGGACAGATGAACGGTGTACA
149	GACCAGGCGCATAGGCTGGCTGACCTTCATCAAGAGTA
150	ATCTTGACAAGAACCGGATATTCATTACCCAAATCAAC
151	GTAACAAAGCTGCTCATTCAGTGAATAAGGCTTGCCCT
152	GACGAGAAACACCAGAACGAGTAGTAAATTGGGCTTGA
153	GATGGTTTAATTTCAACTTTAATCATTGTGAATTACCT
154	TATGCGATTTTAAGAACTGGCTCATTATACCAGTCAGG
155	ACGTTGGGAAGAAAAATCTACGTTAATAAAACGAACTA

156	ACGGAACAACATTATTACAGGTAGAAAGATTCATCAGT
157	TGAGATTTAGGAATACCACATTCAACTAATGCAGATAC
158	ATAACGCCAAAAGGAATTACGAGGCATAGTAAGAGCAA
159	CACTATCATAACCCTCGTTTACCAGACGACGATAAAAA
160	CCAAAATAGCGAGAGGCTTTTGCAAAAGAAGTTTTGCC
161	AGAGGGGGTAATAGTAAAATGTTTAGACTGGATAGCGT
162	CCAATACTGCGGAATCGTCATAAATATTCATTGAATCC
163	CCCTCAAATGCTTTAAACAGTTCAGAAAACGAGAATGA
164	CCATAAATCAAAAATCAGGTCTTTACCCTGACTATTAT
165	AGTCAGAAGCAAAGCGGATTGCATCAAAAAGATTAAGA
166	**GGAAGCCCGATCCTCTTTTGAGGAACAAGTTTTCTTGTAAGACTTCAA**
167	**ATATCGCGTTTCCTCTTTTGAGGAACAAGTTTTCTTGTTTAATTCGAG**
168	**CTTCAAAGCGTCCTCTTTTGAGGAACAAGTTTTCTTGTAACCAGACCG**
169	**GAAGCAAACTTCCTCTTTTGAGGAACAAGTTTTCTTGTCCAACAGGTC**
170	**AGGATTAGAGTCCTCTTTTGAGGAACAAGTTTTCTTGTAGTACCTTTA**
171	**ATTGCTCCTTTCCTCTTTTGAGGAACAAGTTTTCTTGTTTGATAAGAG**
172	**GTCATTTTTGTCCTCTTTTGAGGAACAAGTTTTCTTGTCGGATGGCTT**
173	**AGAGCTTAATTCCTCTTTTGAGGAACAAGTTTTCTTGTTGCTGAATAT**
174	**AATGCTGTAGTCCTCTTTTGAGGAACAAGTTTTCTTGTCTCAACATGT**
175	**TTTAAATATGTCCTCTTTTGAGGAACAAGTTTTCTTGTCAACTAAAGT**
176	**ACGGTGTCTGTCCTCTTTTGAGGAACAAGTTTTCTTGTGAAGTTTCAT**
177	**TCCATATAACTCCTCTTTTGAGGAACAAGTTTTCTTGTAGTTGATTCC**
178	CAATTCTGCGAACGAGTAGATTTAGT
179	TTGACCATTAGATACATTTCGCAAATGGTCAATAACCT
180	GTTTAGCTATATTTTCATTTGGGGCGCGAGCTGAAAAG
181	GTGGCATCAATTCTACTAATAGTAGTAGCATTAACATC
182	CAATAAATCATACAGGCAAGGCAAAGAATTAGCAAAAT
183	TAAGCAATAAAGCCTCAGAGCATAAAGCTAAATCGGTT
184	GTACCAAAAACATTATGACCCTGTAATACTTTTGCGGG
185	AGAAGCCTTTATTTCAACGCAAGGATAAAAATTTTTAG
186	AACCCTCATATATTTTAAATGCAATGCCTGAGTAATGT
187	GTAGGTAAAGATTCAAAAGGGTGAGAAAGGCCGGAGAC
188	AGTCAAATCACCATCAATATGATATTCAACCGTTCTAG
189	CTGATAAATTAATGCCGGAGAGGGTAGCTATTTTTGAG
190	AGATCTACAAAGGCTATCAGGTCATTGCCTGAGAGTCT
191	GGAGCAAACAAGAGAATCGATGAACGGTAATCGTAAAA
192	CTAGCATGTCAATCATATGTACCCCGGTTGATAATCAG
193	AAAAGCCCCAAAAACAGGAAGATTGTATAAGCAAATAT
194	TTAAATTGTAAACGTTAATATTTTGTTAAAATTCGCAT
195	TAAATTTTTGTTAAATCAGCTCATTTTTTAACCAATAG
196	GAACGCCATCAAAAATAATTCGCGTCTGGCCTTCCTGT
197	AGCCAGCTTTCATCAACATTAAATGTGAGCGAGTAACA
198	ACCCGTCGGATTCTCCGTGGGAACAAACGGCGGATTGA

199	CCGTAATGGGATAGGTCACGTTGGTGTAGATGGGCGCA
200	TCGTAACCGTGCATCTGCCAGTTTGAGGGGACGACGAC
201	AGTATCGGCCTCAGGAAGATCGCACTCCAGCCAGCTTT
202	CCGGCACCGCTTCTGGTGCCGGAAACCAGGCAAAGCGC
203	CATTCGCCATTCAGGCTGCGCAACTGTTGGGAAGGGCG
204	ATCGGTGCGGGCCTCTTCGCTATTACGCCAGCTGGCGA
205	AAGGGGGATGTGCTGCAAGGCGATTAAGTTGGGTAACG
206	CCAGGGTTTTCCCAGTCACGACGTTGTAAAACGACGGC
207	CAGTGCCAAGCTTGCATGCCTGCAGGTCGACTCTAGAGGATCTTTT

Table S6.

Sequence of oligonucleotides attached to 1) 0, 2) 5, 3) 10, 4) 20 and 40 kDA PEG used in nanostructure comparing PEG molecular weight (Figure 4a-d).

Oligo number	Sequence (5′→ 3′)
1	GTCTGGGCTGGATGTTGTCG
2	CTGATCCTAGTGACGAAACC-5′5 kDa PEG
3	ACCACTAATGAGTGATATCC-5′10 kDa PEG
4	TGAGGATGTGTAGGTTGAGG-5′20 kDa PEG
5	CGGAAUCAGUGAAUGCUUAUACAUCCGU -5′40 kDa PEG (RNA)

Sequence of DNA oligonucleotide mixture binding 20 and 40 kDA PEG (Figure 4e-f).

Oligo number	Sequence (5′→ 3′)
1	TGAGGATGTGTAGGTTGAGG -5′20 kDa PEG
2	TGAGGATGTGTAGGTTGAGG -5′40 kDa PEG

Table S7.

Sequence of DNA oligonucleotides used for assembly of dsDNA nanostructure used for capture of PEGylated oligonucleotide conjugates.

Oligonucleotide / **Dumbbell** / **Overhangs**

Oligo number	Sequence (5′→ 3′)
1	TTTTCGTAATCATGGTCATAGCTGTTTCCTGTGTGAAATTGTTATC
2	CGCTCACAATTCCACACAACATACGAGCCGGAAGCATA
3	AAGTGTAAAGCCTGGGGTGCCTAATGAGTGAGCTAACT
4	CACATTAATTGCGTTGCGCTCACTGCCCGCTTTCCAGT
5	CGGGAAACCTGTCGTGCCAGCTGCATTAATGAATCGGC
6	CAACGCGCGGGGAGAGGCGGTTTGCGTATTGGGCGCCA
7	GGGTGGTTTTTCTTTTCACCAGTGAGACGGGCAACAGC
8	TGATTGCCCTTCACCGCCTGGCCCTGAGAGAGTTGCAG
9	CAAGCGGTCCACGCTGGTTTGCCCCAGCAGGCGAAAAT
10	CCTGTTTGATGGTGGTTCCGAAATCGGCAAAATCCCTT
11	ATAAATCAAAAGAATAGCCCGAGATAGGGTTGAGTGTT
12	GTTCCAGTTTGGAACAAGAGTCCACTATTAAAGAACGT
13	GGACTCCAACGTCAAAGGGCGAAAAACCGTCTATCAGG
14	GCGATGGCCCACTACGTGAACCATCACCCAAATCAAGT
15	TTTTTGGGGTCGAGGTGCCGTAAAGCACTAAATCGGAA
16	CCCTAAAGGGAGCCCCCGATTTAGAGCTTGACGGGGAA
17	AGCCGGCGAACGTGGCGAGAAAGGAAGGGAAGAAAGCG
18	AAAGGAGCGGGCGCTAGGGCGCTGGCAAGTGTAGCGGT
19	CACGCTGCGCGTAACCACCACACCCGCCGCGCTTAATG
20	CGCCGCTACAGGGCGCGTACTATGGTTGCTTTGACGAG
21	CACGTATAACGTGCTTTCCTCGTTAGAATCAGAGCGGG
22	AGCTAAACAGGAGGCCGATTAAAGGGATTTTAGACAGG
23	AACGGTACGCCAGAATCCTGAGAAGTGTTTTTATAATC
24	AGTGAGGCCACCGAGTAAAAGAGTCTGTCCATCACGCA
25	AATTAACCGTTGTAGCAATACTTCTTTGATTAGTAATA
26	**ACATCACTTGTCCTCTTTTGAGGAACAAGTTTTCTTGTCCTGAGTAGA**
27	**AGAACTCAAATCCTCTTTTGAGGAACAAGTTTTCTTGTCTATCGGCCT**
28	**TGCTGGTAATTCCTCTTTTGAGGAACAAGTTTTCTTGTATCCAGAACA**
29	**ATATTACCGCTCCTCTTTTGAGGAACAAGTTTTCTTGTCAGCCATTGC**
30	**AACAGGAAAATCCTCTTTTGAGGAACAAGTTTTCTTGTACGCTCATGG**
31	**AAATACCTACTCCTCTTTTGAGGAACAAGTTTTCTTGTATTTTGACGC**
32	**TCAATCGTCTTCCTCTTTTGAGGAACAAGTTTTCTTGTGAAATGGATT**

33	**ATTTACATTGTCCTCTTTTGAGGAACAAGTTTCTTGTGCAGATTCAC**
34	**CAGTCACACGTCCTCTTTTGAGGAACAAGTTTCTTGTACCAGTAATA**
35	**AAAGGGACATTCCTCTTTTGAGGAACAAGTTTCTTGTTCTGGCCAAC**
36	**AGAGATAGAATCCTCTTTTGAGGAACAAGTTTCTTGTCCCTTCTGAC**
37	CTGAAAGCGTAAGAATACGTGGCACAGACAATATTTTTGAATGGCT
38	ATTAGTCTTTAATGCGCGAACTGATAGCCCTAAAACAT
39	CGCCATTAAAAATACCGAACGAACCACCAGCAGAAGAT
40	AAAACAGAGGTGAGGCGGTCAGTATTAACACCGCCTGC
41	AACAGTGCCACGCTGAGAGCCAGCAGCAAATGAAAAAT
42	CTAAAGCATCACCTTGCTGAACCTCAAATATCAAACCC
43	TCAATCAATATCTGGTCAGTTGGCAAATCAACAGTTGA
44	AAGGAATTGAGGAAGGTTATCTAAAATATCTTTAGGAG
45	CACTAACAACTAATAGATTAGAGCCGTCAATAGATAAT
46	ACATTTGAGGATTTAGAAGTATTAGACTTTACAAACAA
47	TTCGACAACTCGTATTAAATCCTTTGCCCGAACGTTAT
48	TAATTTTAAAAGTTTGAGTAACATTATCATTTTGCGGA
49	**cgacaacatccagcccagactttttACAAAGAAACCACCAGAAGG**
50	AGCGGAATTATCATCATA
51	TTCCTGATTATCAGATGATGGCAATTCATCAATATAAT
52	CCTGATTGTTTGGATTATACTTCTGAATAATGGAAGGG
53	TTAGAACCTACCATATCAAAATTATTTGCACGTAAAAC
54	AGAAATAAAGAAATTGCGTAGATTTTCAGGTTTAACGT
55	CAGATGAATATACAGTAACAGTACCTTTTACATCGGGA
56	GAAACAATAACGGATTCGCCTGATTGCTTTGAATACCA
57	AGTTACAAAATCGCGCAGAGGCGAATTATTCATTTCAA
58	TTACCTGAGCAAAAGAAGATGATGAAACAAACATCAAG
59	AAAACAAAATTAATTACATTTAACAATTTCATTTGAAT
60	TACCTTTTTTAATGGAAACAGTACATAAATCAATATAT
61	GTGAGTGAATAACCTTGCTTCTGTAAATCGTCGCTATT
62	**ggtttcgtcactaggatcagtttttAGCTTAGATTAAGACGCTGA**
63	GAAGAGTCAATAGTGAAT
64	TTATCAAAATCATAGGTCTGAGAGACTACCTTTTTAAC
65	CTCCGGCTTAGGTTGGGTTATATAACTATATGTAAATG
66	CTGATGCAAATCCAATCGCAAGACAAAGAACGCGAGAA
67	AACTTTTTCAAATATATTTTAGTTAATTTCATCTTCTG
68	ACCTAAATTTAATGGTTTGAAATACCGACCGTGTGATA
69	AATAAGGCGTTAAATAAGAATAAACACCGGAATCATAA
70	TTACTAGAAAAAGCCTGTTTAGTATCATATGCGTTATA
71	CAAATTCTTACCAGTATAAAGCCAACGCTCAACAGTAG
72	GGCTTAATTGAGAATCGCCATATTTAACAACGCCAACA
73	TGTAATTTAGGCAGAGGCATTTTCGAGCCAGTAATAAG
74	AGAATATAAAGTACCGACAAAAGGTAAAGTAATTCTGT
75	**GGATATCACTCATTAGTGGTtttttGAACGCGCCTGTTTATCAAC**

76	AATAGATAAGTCCTGAAC
77	AAGAAAAATAATATCCCATCCTAATTTACGAGCATGTA
78	GAAACCAATCAATAATCGGCTGTCTTTCCTTATCATTC
79	CAAGAACGGGTATTAAACCAAGTACCGCACTCATCGAG
80	AACAAGCAAGCCGTTTTTATTTTCATCGTAGGAATCAT
81	TACCGCGCCCAATAGCAAGCAAATCAGATATAGAAGGC
82	TTATCCGGTATTCTAAGAACGCGAGGCGTTTTAGCGAA
83	CCTCCCGACTTGCGGGAGGTTTTGAAGCCTTAAATCAA
84	GATTAGTTGCTATTTTGCACCCAGCTACAATTTTATCC
85	TGAATCTTACCAACGCTAACGAGCGTCTTTCCAGAGCC
86	TAATTTGCCAGTTACAAAATAAACAGCCATATTATTTA
87	TCCCAATCCAAATAAGAAACGATTTTTGTTTAACGTC
88	**cctcaacctacacatcctcattttttAAAAACAGGGAAGCGCATTA**
89	GACGGGAGAATTAACTGA
90	ACACCCTGAACAAAGTCAGAGGGTAATTGAGCGCTAAT
91	ATCAGAGAGATAACCCACAAGAATTGAGTTAAGCCCAA
92	TAATAAGAGCAAGAAACAATGAAATAGCAATAGCTATC
93	TTACCGAAGCCCTTTTTAAGAAAAGTAAGCAGATAGCC
94	GAACAAAGTTACCAGAAGGAAACCGAGGAAACGCAATA
95	ATAACGGAATACCCAAAAGAACTGGCATGATTAAGACT
96	CCTTATTACGCAGTATGTTAGCAAACGTAGAAAATACA
97	TACATAAAGGTGGCAACATATAAAAGAAACGCAAAGAC
98	ACCACGGAATAAGTTTATTTTGTCACAATCAATAGAAA
99	ATTCATATGGTTTACCAGCGCCAAAGACAAAAGGGCGA
100	CATTCAACCGATTGAGGGAGGGAAGGTAAATATTGACG
101	**CTTGAGCCATTTGGGAATTAttttttACGGATGTATAAGCATTCACTGATTCCG**
102	GAGCCAGCAAAATCACCA
103	GTAGCACCATTACCATTAGCAAGGCCGGAAACGTCACC
104	AATGAAACCATCGATAGCAGCACCGTAATCAGTAGCGA
105	CAGAATCAAGTTTGCCTTTAGCGTCAGACTGTAGCGCG
106	TTTTCATCGGCATTTTCGGTCATAGCCCCCTTATTAGC
107	GTTTGCCATCTTTTCATAATCAAAATCACCGGAACCAG
108	AGCCACCACCGGAACCGCCTCCCTCAGAGCCGCCACCC
109	TCAGAACCGCCACCCTCAGAGCCACCACCCTCAGAGCC
110	GCCACCAGAACCACCACCAGAGCCGCCGCCAGCATTGA
111	CAGGAGGTTGAGGCAGGTCAGACGATTGGCCTTGATAT
112	TCACAAACAAATAAATCCTCATTAAAGCCAGAATGGAA
113	AGCGCAGTCTCTGAATTTACCGTTCCAGTAAGCGTCAT
114	ACATGGCTTTTGATGATACAGGAGTGTACTGGTAATAA
115	**GTTTTAACGGTCCTCTTTTGAGGAACAAGTTTTCTTGTGGTCAGTGCC**
116	**TTGAGTAACATCCTCTTTTGAGGAACAAGTTTTCTTGTGTGCCCGTAT**
117	**AAACAGTTAATCCTCTTTTGAGGAACAAGTTTTCTTGTTGCCCCCTGC**
118	**CTATTTCGGATCCTCTTTTGAGGAACAAGTTTTCTTGTACCTATTATT**

119	**CTGAAACATGTCCTCTTTTGAGGAACAAGTTTTCTTGTAAAGTATTAA**
120	**GAGGCTGAGATCCTCTTTTGAGGAACAAGTTTTCTTGTCTCCTCAAGA**
121	**GAAGGATTAGTCCTCTTTTGAGGAACAAGTTTTCTTGTGATTAGCGGG**
122	**GTTTTGCTCATCCTCTTTTGAGGAACAAGTTTTCTTGTGTACCAGGCG**
123	**GATAAGTGCCTCCTCTTTTGAGGAACAAGTTTTCTTGTGTCGAGAGGG**
124	**TTGATATAAGTCCTCTTTTGAGGAACAAGTTTTCTTGTTATAGCCCGG**
125	**AATAGGTGTATCCTCTTTTGAGGAACAAGTTTTCTTGTTCACCGTACT**
126	CAGGAGGTTTAGTACCGCCACCCTCAGAACCGCCACCCTCAGAACC
127	GCCACCCTCAGAGCCACCACCCTCATTTTCAGGGATAG
128	CAAGCCCAATAGGAACCCATGTACCGTAACACTGAGTT
129	TCGTCACCAGTACAAACTACAACGCCTGTAGCATTCCA
130	CAGACAGCCCTCATAGTTAGCGTAACGATCTAAAGTTT
131	TGTCGTCTTTCCAGACGTTAGTAAATGAATTTTCTGTA
132	TGGGATTTTGCTAAACAACTTTCAACAGTTTCAGCGGA
133	GTGAGAATAGAAAGGAACAACTAAAGGAATTGCGAATA
134	ATAATTTTTTCACGTTGAAAATCTCCAAAAAAAAGGCT
135	CCAAAAGGAGCCTTTAATTGTATCGGTTTATCAGCTTG
136	CTTTCGAGGTGAATTTCTTAAACAGCTTGATACCGATA
137	GTTGCGCCGACAATGACAACAACCATCGCCCACGCATA
138	ACCGATATATTCGGTCGCTGAGGCTTGCAGGGAGTTAA
139	AGGCCGCTTTTGCGGGATCGTCACCCTCAGCAGCGAAA
140	GACAGCATCGGAACGAGGGTAGCAACGGCTACAGAGGC
141	TTTGAGGACTAAAGACTTTTTCATGAGGAAGTTTCCAT
142	TAAACGGGTAAAATACGTAATGCCACTACGAAGGCACC
143	AACCTAAAACGAAAGAGGCAAAAGAATACACTAAAACA
144	CTCATCTTTGACCCCCAGCGATTATACCAAGCGCGAAA
145	CAAAGTACAACGGAGATTTGTATCATCGCCTGATAAAT
146	TGTGTCGAAATCCGCGACCTGCTCCATGTTACTTAGCC
147	GGAACGAGGCGCAGACGGTCAATCATAAGGGAACCGAA
148	CTGACCAACTTTGAAAGAGGACAGATGAACGGTGTACA
149	GACCAGGCGCATAGGCTGGCTGACCTTCATCAAGAGTA
150	ATCTTGACAAGAACCGGATATTCATTACCCAAATCAAC
151	GTAACAAAGCTGCTCATTCAGTGAATAAGGCTTGCCCT
152	GACGAGAAACACCAGAACGAGTAGTAAATTGGGCTTGA
153	GATGGTTTAATTTCAACTTTAATCATTGTGAATTACCT
154	TATGCGATTTTAAGAACTGGCTCATTATACCAGTCAGG
155	ACGTTGGGAAGAAAAATCTACGTTAATAAAACGAACTA
156	ACGGAACAACATTATTACAGGTAGAAAGATTCATCAGT
157	TGAGATTTAGGAATACCACATTCAACTAATGCAGATAC
158	ATAACGCCAAAAGGAATTACGAGGCATAGTAAGAGCAA
159	CACTATCATAACCCTCGTTTACCAGACGACGATAAAAA
160	CCAAAATAGCGAGAGGCTTTTGCAAAAGAAGTTTTGCC
161	AGAGGGGGTAATAGTAAAATGTTTAGACTGGATAGCGT

162	CCAATACTGCGGAATCGTCATAAATATTCATTGAATCC
163	CCCTCAAATGCTTTAAACAGTTCAGAAAACGAGAATGA
164	CCATAAATCAAAAATCAGGTCTTTACCCTGACTATTAT
165	AGTCAGAAGCAAAGCGGATTGCATCAAAAAGATTAAGA
166	GGAAGCCCGAAAGACTTCAAATATCGCGTTTTAATTCG
167	AGCTTCAAAGCGAACCAGACCGGAAGCAAACTCCAACA
168	GGTCAGGATTAGAGAGTACCTTTAATTGCTCCTTTTGA
169	TAAGAGGTCATTTTTGCGGATGGCTTAGAGCTTAATTG
170	CTGAATATAATGCTGTAGCTCAACATGTTTTAAATATG
171	CAACTAAAGTACGGTGTCTGGAAGTTTCATTCCATATA
172	ACAGTTGATTCCCAATTCTGCGAACGAGTAGATTTAGT
173	TTGACCATTAGATACATTTCGCAAATGGTCAATAACCT
174	GTTTAGCTATATTTTCATTTGGGGCGCGAGCTGAAAAG
175	GTGGCATCAATTCTACTAATAGTAGTAGCATTAACATC
176	CAATAAATCATACAGGCAAGGCAAAGAATTAGCAAAAT
177	TAAGCAATAAAGCCTCAGAGCATAAAGCTAAATCGGTT
178	GTACCAAAAACATTATGACCCTGTAATACTTTTGCGGG
179	AGAAGCCTTTATTTCAACGCAAGGATAAAAATTTTTAG
180	AACCCTCATATATTTTAAATGCAATGCCTGAGTAATGT
181	GTAGGTAAAGATTCAAAAGGGTGAGAAAGGCCGGAGAC
182	AGTCAAATCACCATCAATATGATATTCAACCGTTCTAG
183	CTGATAAATTAATGCCGGAGAGGGTAGCTATTTTTGAG
184	AGATCTACAAAGGCTATCAGGTCATTGCCTGAGAGTCT
185	GGAGCAAACAAGAGAATCGATGAACGGTAATCGTAAAA
186	CTAGCATGTCAATCATATGTACCCCGGTTGATAATCAG
187	AAAAGCCCCAAAAACAGGAAGATTGTATAAGCAAATAT
188	TTAAATTGTAAACGTTAATATTTTGTTAAAATTCGCAT
189	TAAATTTTTGTTAAATCAGCTCATTTTTTAACCAATAG
190	GAACGCCATCAAAAATAATTCGCGTCTGGCCTTCCTGT
191	AGCCAGCTTTCATCAACATTAAATGTGAGCGAGTAACA
192	ACCCGTCGGATTCTCCGTGGGAACAAACGGCGGATTGA
193	CCGTAATGGGATAGGTCACGTTGGTGTAGATGGGCGCA
194	TCGTAACCGTGCATCTGCCAGTTTGAGGGGACGACGAC
195	AGTATCGGCCTCAGGAAGATCGCACTCCAGCCAGCTTT
196	CCGGCACCGCTTCTGGTGCCGGAAACCAGGCAAAGCGC
197	CATTCGCCATTCAGGCTGCGCAACTGTTGGGAAGGGCG
198	ATCGGTGCGGGCCTCTTCGCTATTACGCCAGCTGGCGA
199	AAGGGGGATGTGCTGCAAGGCGATTAAGTTGGGTAACG
200	CCAGGGTTTTCCCAGTCACGACGTTGTAAAACGACGGC
201	CAGTGCCAAGCTTGCATGCCTGCAGGTCGACTCTAGAGGATCTTTT

Table S8.

Statistical analysis for current spike observables in **Figures 4c-4d**, including descriptive statistics, Shapiro-Wlik test, Levene's test, OMNIBUS Test, and post-hoc Games-Howell pairwise comparisons.

Figure 4c

Descriptive Statistics

Group	n	Mean	Std	Median	Min	Max
0 kDa	194	0.1168	0.0511	0.1061	0.0217	0.3642
5 kDa	194	0.1756	0.0613	0.1832	0.0276	0.3371
10 kDa	194	0.2191	0.0776	0.2306	0.0360	0.4712
20 kDa	194	0.2409	0.1009	0.2653	0.0335	0.5372
40 kDa	194	0.4317	0.1422	0.4702	0.0367	0.6521

Normality: Shapiro-Wilk test (H_0: data are normal)

Group	W	p-value	Normality
0 kDa	0.9256	0.0000	No
5 kDa	0.9870	0.0712	Yes
10 kDa	0.9757	0.0019	No
20 kDa	0.9133	0.0000	No
40 kDa	0.8637	0.0000	No

→ All groups normal: False

Homogeneity of variance: Levene's test (H_0: variances equal)

- Levene statistic: 24.8335
- p-value: 0.0000 ***
- Equal variances: False

OMNIBUS Test

- **Welch's ANOVA**
  $F(4, 471.1300) = 255.1412$, $p = 0.000000$ ***
  Significant: True
- **Standard ANOVA (for reference)**
  $F = 319.8750$, $p = 0.000000$ ***
- **Kruskal-Wallis (non-parametric)**
  $H = 453.3789$, $p = 0.000000$ ***
  Significant: True

Post-hoc — Games-Howell pairwise comparisons

Pair	D	SE	q	df	p-value
0-5	-0.0588	0.0057	14.5155	374.0970	0.0000
0-10	-0.1023	0.0067	21.6725	333.9870	0.0000
0-20	-0.1241	0.0081	21.6080	285.9970	0.0000
0-40	-0.3149	0.0108	41.0480	242.1309	0.0000
5-10	-0.0435	0.0071	8.6613	366.1833	0.0000
5-20	-0.0653	0.0085	10.8975	318.1897	0.0000
5-40	-0.2561	0.0111	32.5812	262.2579	0.0000
10-20	-0.0218	0.0091	3.3777	362.1608	0.0072
10-40	-0.2126	0.0116	25.8497	298.6876	0.0000
20-40	-0.1908	0.0125	21.5496	348.1219	0.0000

Summary

- Groups compared: 5
  Equal variances: False (Levene p=0.0000)
  All groups normal: False

- Suitable test: Welch's ANOVA
  Suitable post-hoc: Games-Howell

- Welch's ANOVA: F=255.1412., p=0.000000 ***
  Kruskal-Wallis: H=255.1412, p=0.000000 ***

Figure 4d

Descriptive Statistics

Group	n	Mean	Std	Median	Min	Max
0 kDa	194	4.81	1.68	4.56	2.09	11.41
5 kDa	194	6.50	2.01	6.59	2.04	11.07
10 kDa	194	7.31	2.45	7.05	2.37	20.04
20 kDa	194	9.24	3.56	9.23	2.14	23.32
40 kDa	194	13.20	3.88	13.80	2.63	26.39

Normality: Shapiro-Wilk test (H_0: data are normal)

Group	W	p-value	Normality
0 kDa	0.9503	0.0000	No
5 kDa	0.9906	0.2404	Yes
10 kDa	0.9505	0.0000	No
20 kDa	0.9809	0.0095	No
40 kDa	0.9599	0.0000	No

→ All groups normal: False

Homogeneity of variance: Levene's test (H_0: variances equal)

- Levene statistic: 26.9254
- p-value: 0.0000 ***
- Equal variances: False

OMNIBUS Test

- **Welch's ANOVA**
  $F(4, 472.24) = 224.2523$, $p = 0.000000$ ***
  Significant: True
- **Standard ANOVA (for reference)**
  $F = 245.5839$, $p = 0.000000$ ***
- **Kruskal-Wallis (non-parametric)**
  $H = 451.9016$, $p = 0.000000$ ***
  Significant: True

Post-hoc — Games-Howell pairwise comparisons

Pair	D	SE	q	df	p-value
0-5	-1.7022	0.1885	12.7706	374.4255	0.0000
0-10	-2.5075	0.2138	16.5839	341.8132	0.0000
0-20	-4.4430	0.2834	22.1744	275.0080	0.0000
0-40	-8.3949	0.3041	39.0427	263.1407	0.0000
5-10	-0.8053	0.2280	4.9959	371.7029	0.0000
5-20	-2.7409	0.2942	13.1762	304.5259	0.0000
5-40	-6.6927	0.3142	30.1253	289.6456	0.0000
10-20	-1.9355	0.3110	8.8008	342.2837	0.0000
10-40	-5.8874	0.3300	25.2297	325.9556	0.0000
20-40	-3.9519	0.3788	14.7546	383.2421	0.0000

Summary

- Groups compared: 5
  Equal variances: False (Levene p=0.0000)
  All groups normal: False

- Suitable test: Welch's ANOVA
  Suitable post-hoc: Games-Howell

- Welch's ANOVA: F=224.2523, p=0.000000 ***
  Kruskal-Wallis: H=451.9016, p=0.000000 ***

Table S9.

The table includes the IV curves of the nanopores used to characterize the DNA nanostructures prior to the measurement. The ionic current and RMS noise at 600 mV is presented for each pore presented in the Main text.

Pore number	Sample	Current, RMS noise at 600 mV	Current / voltage curve (IV curve)
1	DNA nanostructures 'P' and 'R' (Figure 1 and Figure 2a-c)	12.6 nA<br>6.1 pA	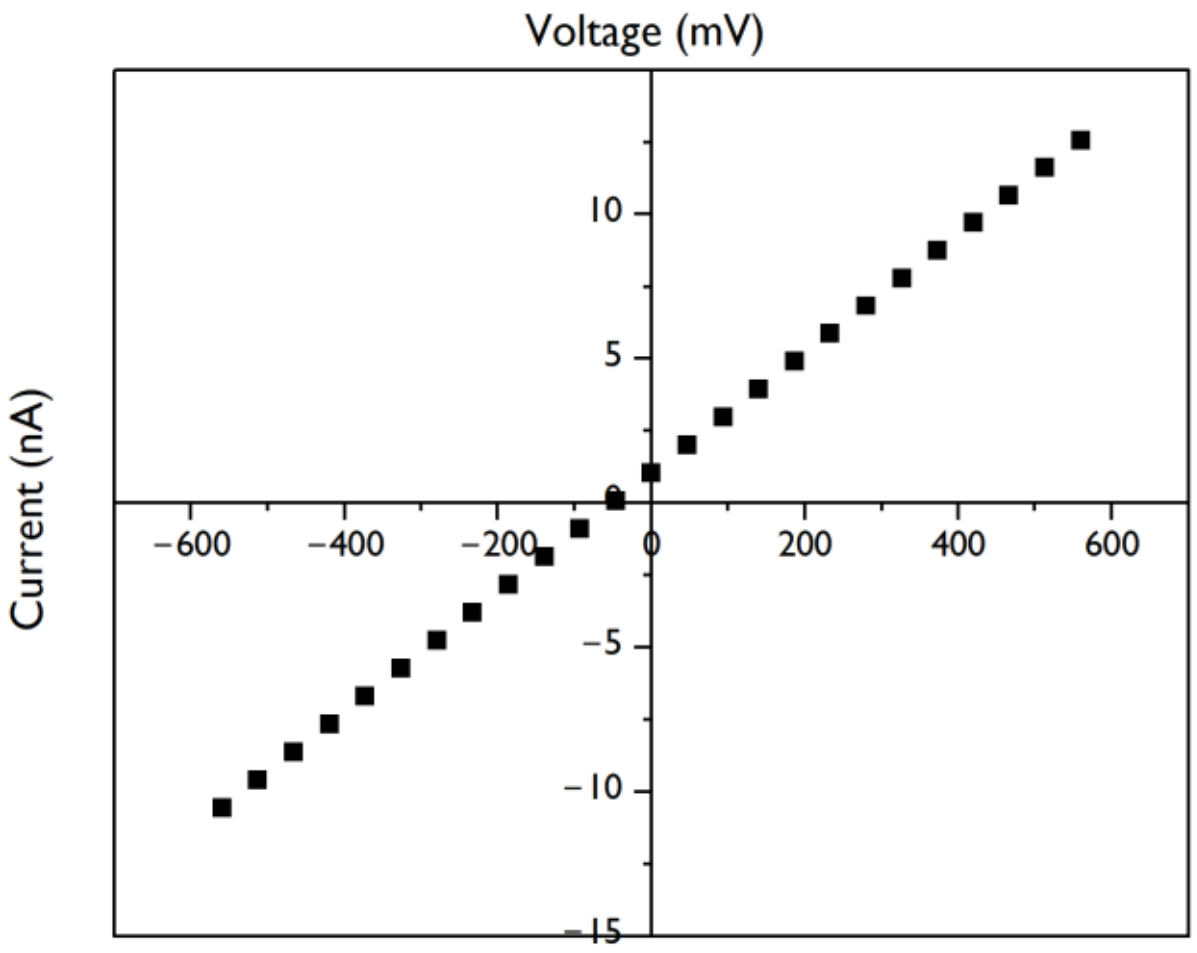
2	DNA nanostructure 'P' in horse serum (Figure 2d-f)	8.4 nA<br>6.3 pA	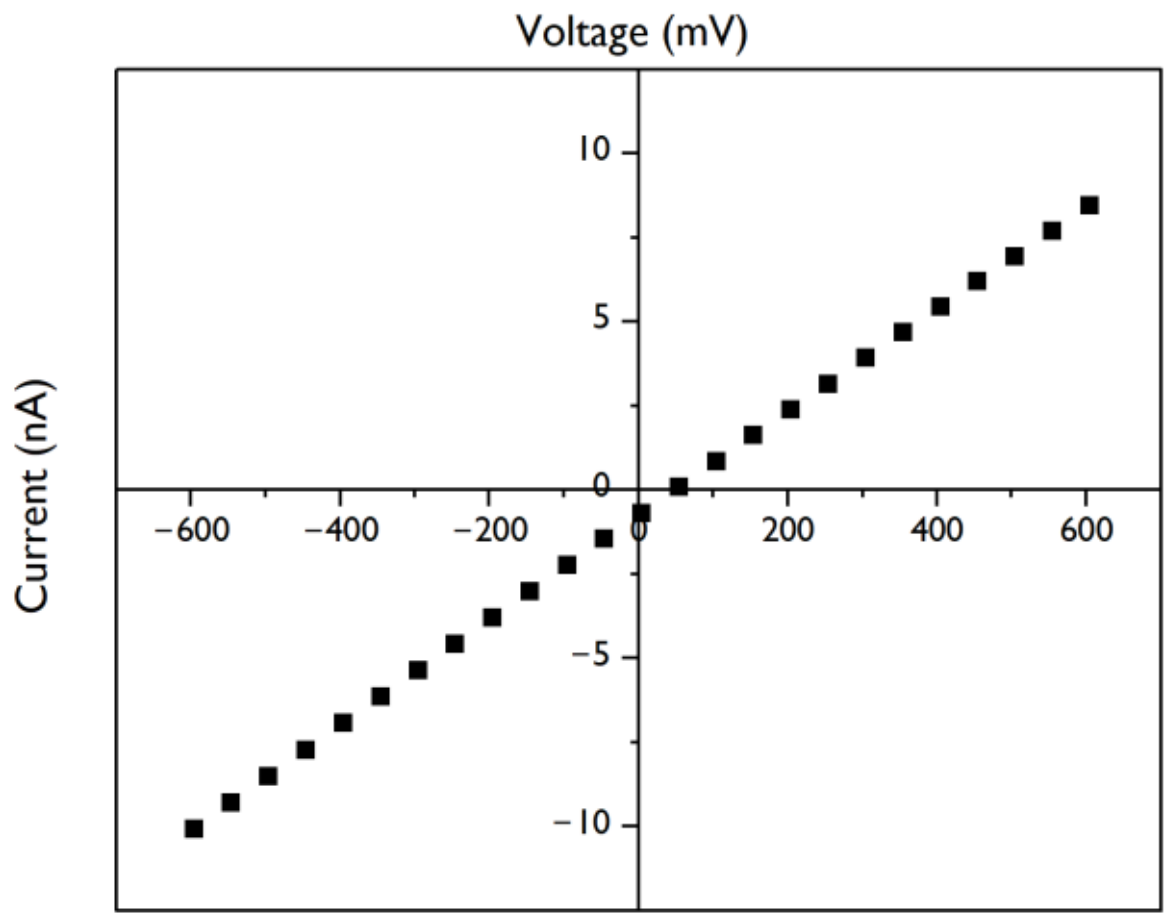

3	DNA nanostructure 'S' (Figure 3a-d)	7.7 nA<br>6.5 pA	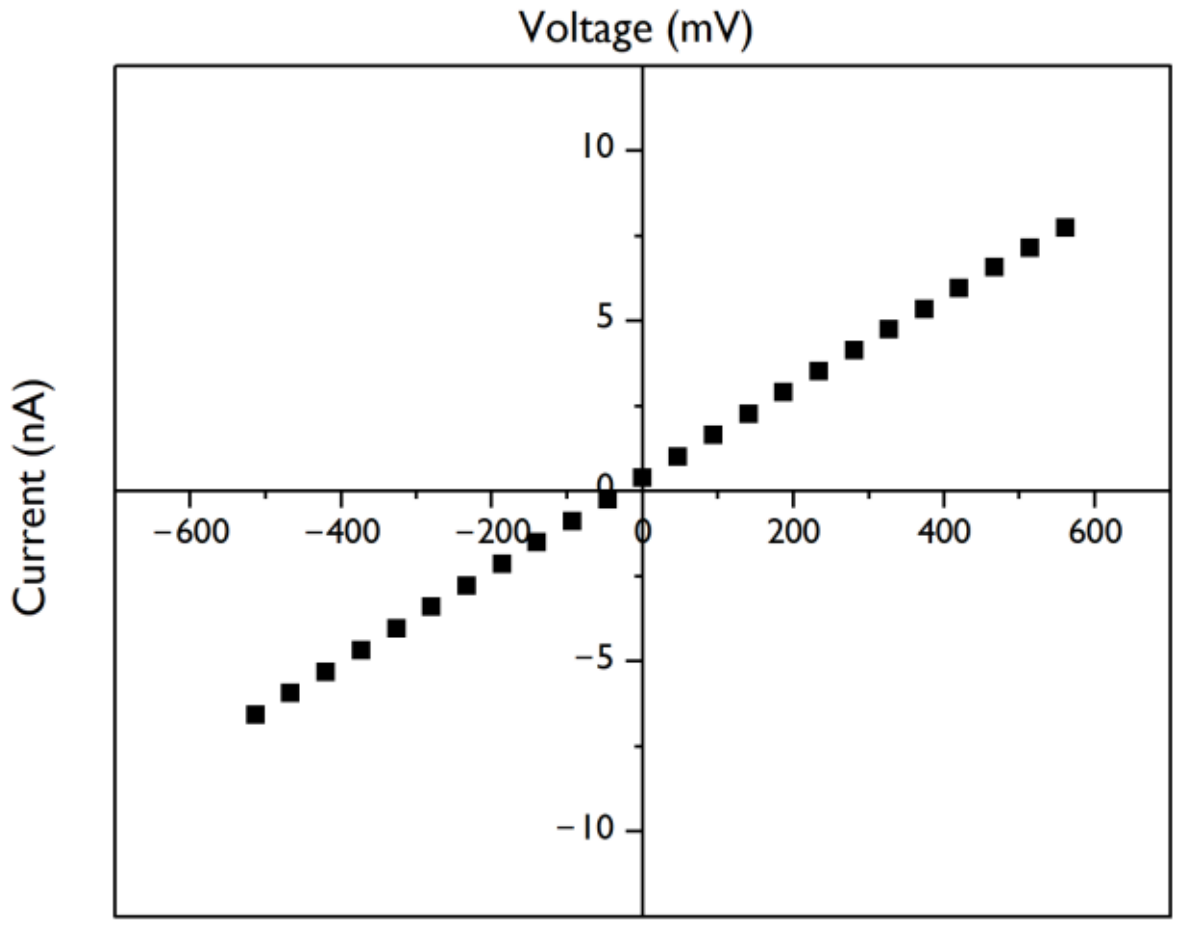
4	DNA nanostructure 'S' and 'P' 5min in horse serum (Figure 3e)	9.1 nA<br>5.8 pA	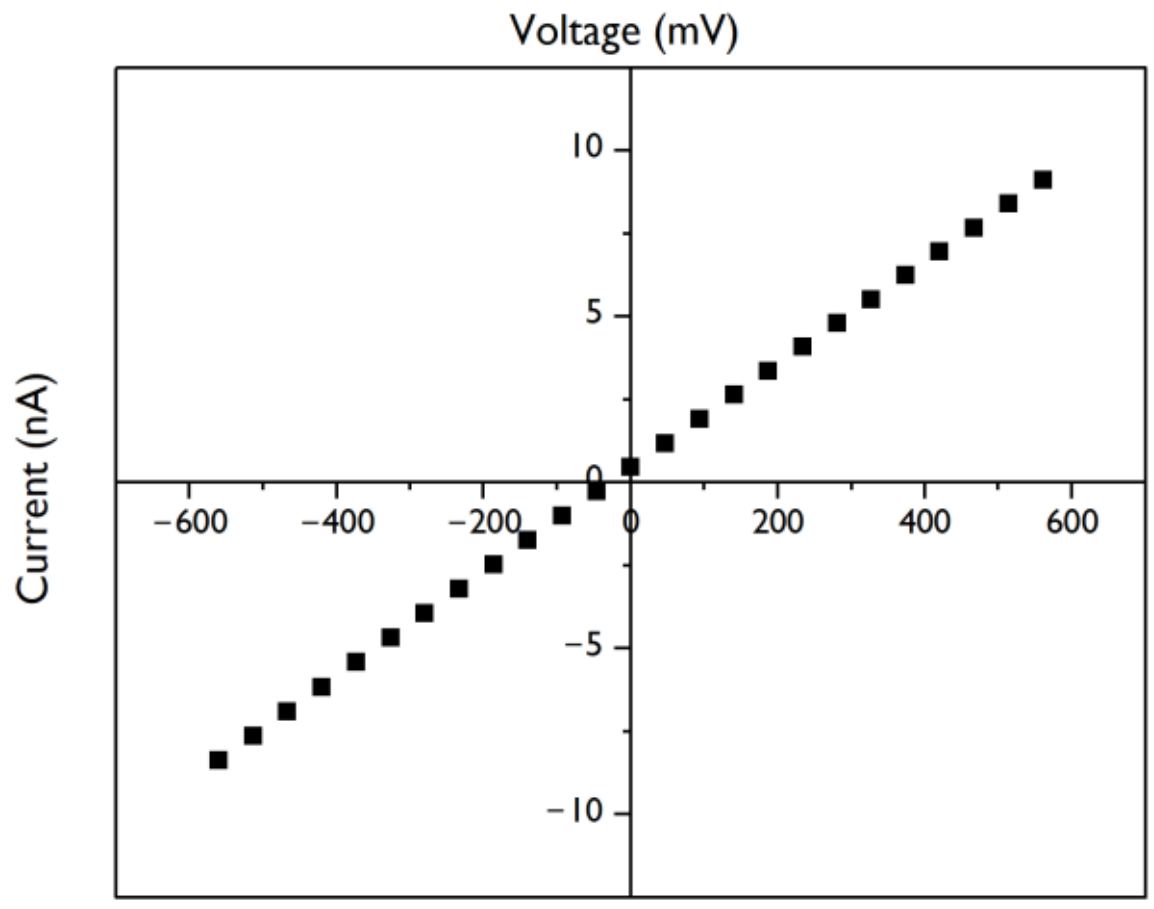
5	DNA nanostructure 'S' and 'P' 24 h in horse serum (Figure 3f)	12.9 nA<br>6.4 pA	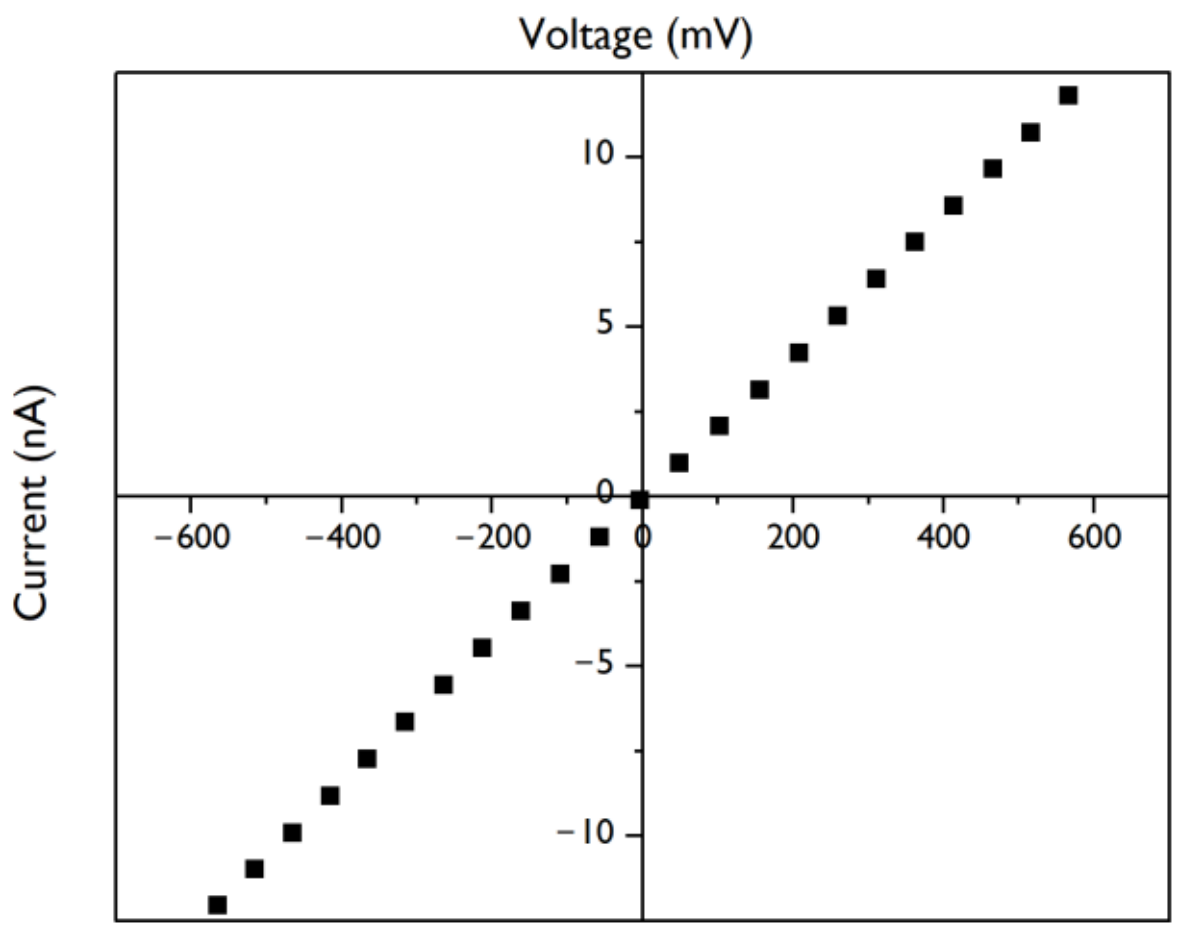

6	DNA nanostructure for 0,5, 10, 20 kDa PEG and competing capture nanostructure (Figure 4)	8.1 nA<br>6.4 pA	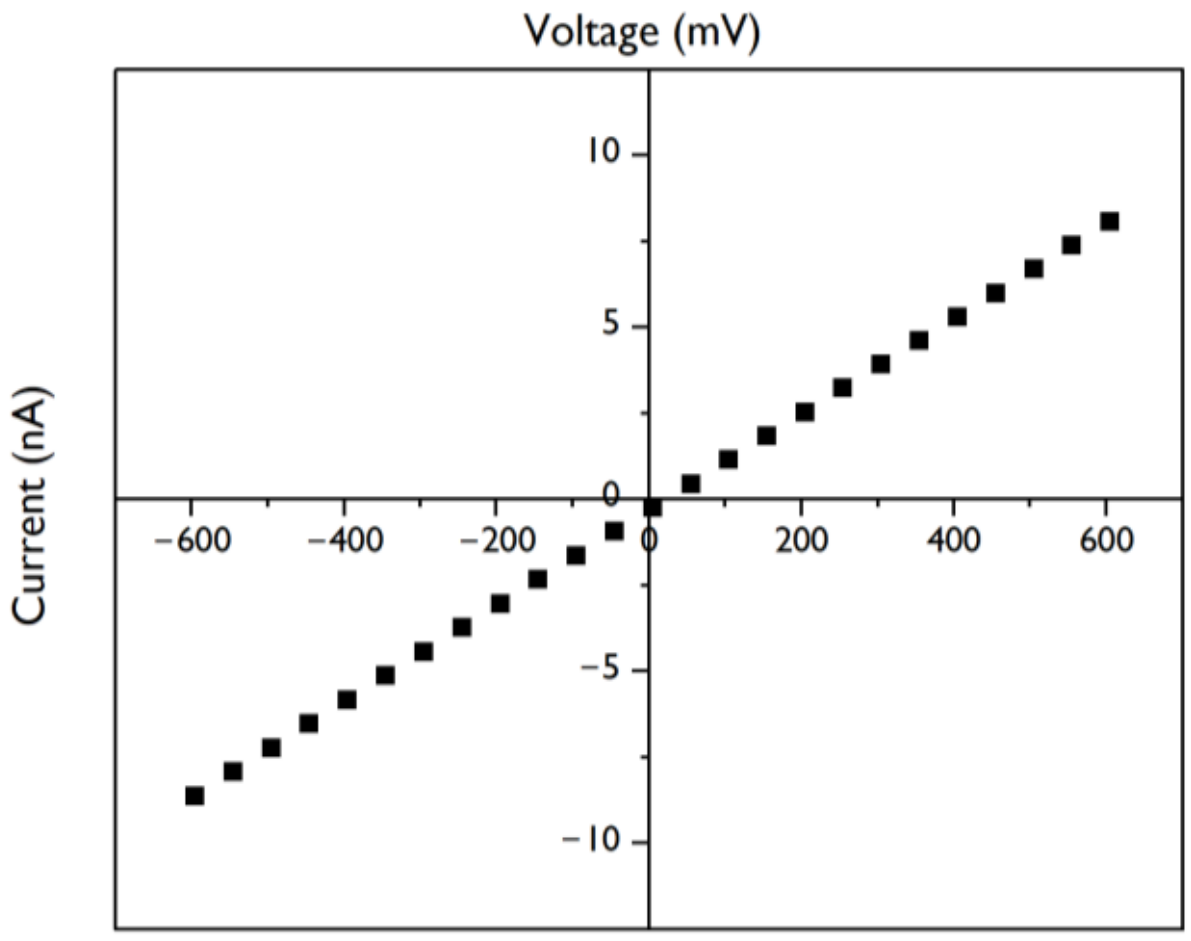
